# Associations of co-exposure to metals and polycyclic aromatic hydrocarbons with aging biomarkers: insights from epidemiology and network toxicology

**DOI:** 10.3389/fpubh.2026.1844457

**Published:** 2026-05-28

**Authors:** Min Mu, Shikai Xiong, Xin Guan, Liliang Yu, Huan Guo, Xuefeng Lai

**Affiliations:** 1 Key Laboratory of Industrial Dust Prevention and Control and Occupational Health and Safety, Ministry of Education, Anhui University of Science and Technology, Huainan, Anhui, China; 2 Provincial Key Laboratory of Occupational Health, School of Public Health, Healthy Hubei Development and Social Progress Research Center of the Key Research Base of Humanities and Social Sciences in Hubei Province, Wuhan University of Science and Technology, Wuhan, Hubei, China; 3 Department of Occupational and Environmental Health, Ministry of Education Key Laboratory of Environment and Health, School of Public Health, Tongji Medical College, Huazhong University of Science and Technology, Wuhan, Hubei, China

**Keywords:** co-exposure, cohort study, metals, MtDNAcn, polycyclic aromatic hydrocarbons, telomere shortening

## Abstract

The impact of polycyclic aromatic hydrocarbons (PAHs) and metals on aging biomarkers-telomere length (TL) and mitochondrial DNA copy number (mtDNAcn)- has been investigated separately. However, their combined effects, particularly in highly exposed occupational populations, remain unexamined, despite their frequent co-occurrence in environmental settings. This study measured baseline urinary and blood samples from 867 coke oven workers at baseline for 33 chemical pollutants and mtDNAcn, and assessed leukocyte TL at both baseline and follow-up. The combined effect of metals and PAHs on aging biomarkers was evaluated using weighted quantum sum (WQS) regression and Bayesian kernel machine regression (BKMR), and mediation analyses were also conducted. Multiple linear regression incorporating 33 pollutants and least absolute shrinkage and selection operator (LASSO) regression were used to identify individual exposures with significant effects, while restricted cubic splines were applied to explore potential nonlinear dose–response relationships. We observed that both the WQS and BKMR indicated that 33 chemical mixtures exposure was significantly negatively associated with the TL ratio (follow-up/baseline) and positively associated with baseline mtDNAcn. Urinary 1-hydroxypyrene, molybdenum, selenium (Se), and barium were identified as the major contributors. Furthermore, oxidative stress and inflammation mediated the link of major individual pollutants as well as the chemical mixture to biological aging. Network toxicology analysis identified *HSP90AA1* and *HSP90AB1* as core molecular targets underlying the effect of Se on TL regulation. To our knowledge, this is the first study to investigate the joint effects and underlying mechanisms of co-exposure to PAHs and metals on biomarkers of biological aging.

## Highlights

- Common occupational exposure to PAHs and metals was dose-responsively associated with accelerated telomere attrition and increased mtDNA copy number.
- 1-Hydroxypyrene, Mo, Se, and Ba were identified as the primary chemicals associated with the changes in aging biomarkers.
- Oxidative stress and inflammation mediated the associations of individual pollutants and the chemical mixture with biological aging.
- *HSP90AA1* and *HSP90AB1* were pinpointed as core molecular targets linking Se to telomere length regulation.

## 1 Introduction

Rapid industrialization and urbanization have led to a range of environmental pollution issues caused by toxic chemicals. Metals and polycyclic aromatic hydrocarbons (PAHs) are major components of such pollutants, widely distributed across human environments and contributing to significant adverse health effects (1–3). Furthermore, PAHs and metals are frequently found to co-occur at substantial levels in common pollution sources, including particulate matter and contaminated food and water (4, 5). At elevated concentrations, this co-exposure may lead to synergistic interactions in the environment, resulting in greater health risks compared to single-pollutant exposure (6, 7). Consequently, there is growing scientific interest in the health effects and underlying mechanisms of co-exposure to these chemical classes (5, 8, 9).

Aging is a complex process characterized by multisystem physiological decline and is a key risk factor for chronic diseases and mortality (10). Telomeres, recognized as crucial markers of human aging, play significant roles in the pathogenesis of conditions such as cancer, diabetes, and cardiovascular diseases (11). In light of this, previous studies have investigated the associations between exposure to individual pollutants (metals or PAHs) and molecular biomarkers of aging, including telomere length (TL) and mitochondrial DNA copy number (mtDNAcn) (10–14). However, these exposures not only commonly co-occur in both general living and occupational environments, but also present at substantially higher concentrations with potentially more pronounced interactive effects in occupational populations, such as coke oven workers (15, 16). Therefore, further investigation into the association between combined high-level exposure to metals and PAHs and biomarkers of aging is warranted.

Previous studies, including from our laboratory, on single pollutants have demonstrated that both metals and PAHs can induce similar early-stage adverse effects, such as oxidative stress and inflammation, which may ultimately lead to genetic damage (14, 17–19). Notably, synergistic interactions between metals, redox-active quinones, and PAHs can generate reactive oxygen species, thereby exacerbating oxidative stress (15, 20). In this study, we further investigated the mediating roles of oxidative stress and inflammation in the associations of both individual and mixed pollutant exposures with aging biomarkers. Additionally, we employed a network toxicology approach to systematically characterize the potential toxicological pathways and molecular mechanisms by which metals and PAHs may influence telomere regulation.

In this longitudinal cohort study of 867 coke oven workers, we measured baseline concentrations of 22 metals and 11 PAHs metabolites, mtDNAcn, and leukocyte TL at both baseline and follow-up. We aimed to elucidate the combined effect and potential interactions of co-exposure to these 33 chemicals on biological aging. Subsequently, we examined the mediating effects of oxidative stress and inflammation in these associations, along with the potential toxicological pathways and molecular mechanisms involved.

## 2 Methods

### 2.1 Study population

In October 2010, a total of 1,628 workers who had been employed for over 1 year at a coke oven plant in Wuhan, China, were enrolled in this study. By the follow-up in October 2014, 1,243 participants completed the survey, yielding a follow-up rate of 76.4%. All participants were unrelated Han Chinese residents from the same urban district of Wuhan. Trained research staff from our team administered face-to-face interviews using a standardized questionnaire to collect information on general demographics (age, sex, and education level), lifestyle habits (smoking, alcohol consumption, and physical activity), health status, and occupational exposure history. For this study, a current smoker was defined as an individual who smoked at least one cigarette per day for more than 1 year. Similarly, a current drinker was defined as someone who consumed alcohol at least once per week for more than 1 year. An exerciser was defined as an individual who engaged in physical activity for the purpose of exercise more than three times per week, with each session lasting over 20 min.

From the initial cohort, we excluded the following participants to ensure data completeness: 47 individuals with missing blood samples, 16 who self-reported a history of coronary heart disease, 120 with incomplete data on peripheral blood mtDNAcn, and 127 with incomplete baseline data on urinary concentrations of 22 metals, 10 PAHs metabolites, or plasma concentrations of 7,8-dihydrodiol-9,10-epoxide-albumin (BPDE-Alb) adducts. A further 66 participants were excluded due to missing covariate data. Consequently, the final analytical sample comprised 867 participants (Supplementary Figure S1).

### 2.2 Determination of polycyclic aromatic hydrocarbons exposure biomarkers

The baseline urinary levels of twelve PAH metabolites—1-hydroxynaphthalene (1-OHNa), 2-hydroxynaphthalene (2-OHNa), 2-hydroxyfluorene (2-OHFlu), 9-hydroxyfluorene (9-OHFlu), 1-hydroxyphenanthrene (1-OHPh), 2-hydroxyphenanthrene (2-OHPh), 3-hydroxyphenanthrene (3-OHPh), 4-hydroxyphenanthrene (4-OHPh), 9-hydroxyphenanthrene (9-OHPh), 1-hydroxypyrene (1-OHP), 3-hydroxybenzo[a]pyrene (3-OHBaP) and 6-hydroxychrysene (6-OHChr)—were quantified with an Agilent 5975B/6890 N GC–MS system (Agilent, CA, USA) following NIOSH Method 5,506. Method limits of detection (LODs) ranged from 0.1 μg/L to 1.4 μg/L; values below the LOD were replaced by LOD/2. Because 6-OHChr and 3-OHBaP were detected in <10% of samples, only the remaining ten metabolites (detection rate >90%) were retained as internal exposure biomarkers of non-carcinogenic PAHs. After creatinine adjustment, concentrations were expressed as μg/mmol creatinine, and their sum was computed as ΣOH-PAHs.

The ten OH-PAHs outlined above derive from non-carcinogenic parent PAHs. To capture the full spectrum of internal exposure, we also quantified the albumin adduct of (+)-anti-benzo[a]pyrene-7,8-diol-9,10-epoxide. B[a]P, a prototypic carcinogenic PAH, is metabolically activated to BPDE, which covalently binds plasma albumin and distributes systemically. Adduct concentrations were determined in duplicate by a validated ELISA protocol, and the mean of the two measurements is reported as ng/mg albumin.

### 2.3 Determination of urinary metals

Urinary concentrations of 23 metal elements—specifically aluminum (Al), antimony (Sb), arsenic (As), barium (Ba), cadmium (Cd), chromium (Cr), cobalt (Co), copper (Cu), iron (Fe), lead (Pb), manganese (Mn), molybdenum (Mo), nickel (Ni), rubidium (Rb), selenium (Se), strontium (Sr), thallium (Tl), tin (Sn), titanium (Ti), tungsten (W), uranium (U), vanadium (V), and zinc (Zn)—were determined using inductively coupled plasma mass spectrometry (ICP-MS; 7,700X series, Agilent Technologies). U was subsequently excluded from further analysis as its concentration fell below the limit of quantification (LOQ) in over 80% of the samples. To account for urine dilution, all metal concentrations were standardized by urinary creatinine levels and are expressed as μg per mmol creatinine.

### 2.4 Determination of aging biomarkers

Peripheral leukocyte TL and mtDNAcn in peripheral blood samples from coke oven workers were analyzed using real-time quantitative polymerase chain reaction (qPCR). For TL measurement, genomic DNA was extracted using the Gentra Puregene kit. After quantifying DNA concentration with a Nanodrop spectrophotometer and verifying integrity by gel electrophoresis, the relative telomere length was determined as the ratio (T/S) of the telomere repeat copy number to a single-copy gene (HBG) on an ABI Prism 7,900 HT system using the SYBR Green method. For mtDNAcn analysis, DNA was extracted using a magnetic nanoparticle-based method. Purity was assessed spectrophotometrically, and relative mtDNAcn was quantified using a Light Cycler system with a SYBR Green I kit, normalized to the nuclear reference gene HBG. Detailed protocols for both assays have been described in our previous study (14, 21). To ensure inter-assay consistency, standard curves were generated using serially diluted pooled reference DNA samples in each experiment.

### 2.5 Assessment of oxidative stress and inflammatory biomarkers

Urinary concentrations of 8-hydroxy-2′-deoxyguanosine (8-OHdG) were measured by high-performance liquid chromatography with electrochemical detection, and 8-iso-prostaglandin F2α (8-iso-PGF2α) was quantified using a commercial enzyme-linked immunosorbent assay (ELISA) kit (17). Both biomarkers were adjusted for urinary creatinine levels. Given prior evidence linking 8-OHdG, but not 8-iso-PGF2α, to telomere attrition, the present analysis focused primarily on 8-OHdG as the key oxidative stress marker. Additionally, a novel integrated inflammatory index, the Systemic Inflammation Response Index (SIRI), was calculated from complete blood count data using the following formula: SIRI = (neutrophil count × monocyte count) / lymphocyte count.

### 2.6 Statistical analysis

To approximate a normal distribution for subsequent analyses, the concentrations of PAHs, metals, and 8-OHdG were natural-log (ln) transformed. This transformation was applied to better meet model assumptions and mitigate the influence of outliers. Additionally, Pearson correlation coefficients were calculated to assess the interrelationships among all ln-transformed pollutant concentrations (Supplementary Figure S2). The change in TL (expressed as the TL-ratio) was calculated as the ln-transformed ratio of follow-up to baseline relative telomere length in peripheral blood leukocytes: TL-ratio = ln (follow-up TL/baseline TL) = ln (follow-up TL)−ln (baseline TL). This metric offers three advantages: (i) it uses each participant’s baseline value as an internal reference, thereby removing between-individual differences and capturing relative change; (ii) the ln-transformation compresses the originally right-skewed distribution of telomere length toward normality, satisfying model assumptions, while the ratio remains strictly positive, preventing undefined logarithms (Supplementary Figure S3); (iii) the model coefficient β can be directly interpreted as a β × 100% change in relative telomere length for each 1% increase in the concentration of the mixture component.

The associations between exposure to chemical mixtures and aging biomarkers were assessed using a series of analytical approaches. Multivariable linear regression models incorporating all 33 chemicals simultaneously were first constructed to estimate the effect of individual contaminants with mutual adjustment for co-exposures. Potential non-linear dose–response relationships between exposures (metals and PAHs) and aging biomarkers were examined using restricted cubic spline (RCS) regression models with 4 knots, with the median exposure level set as the reference point. The overall effect of the chemical mixture was evaluated using Weighted Quantile Sum (WQS) regression (22). Key contributors and their potential interactions within the mixture were further investigated using the least absolute shrinkage and selection operator (LASSO) penalized regression and stratified Bayesian kernel machine regression (BKMR) models. The optimal number of exposure groups was first determined using the elbow method, followed by specific group assignment via hierarchical clustering (Supplementary Figure S4). Causal mediation analysis based on multivariable linear regression frameworks was conducted to explore two potential biological pathways (oxidative stress and inflammation) through which the chemical mixture might influence biological aging. This analysis quantified the direct and indirect effects and estimated the proportion mediated. 1,000 times quasi-Bayesian Monte Carlo simulation was developed to calculate the 95% CIs of the proportion mediated. To assess the mediating roles of oxidative stress and inflammatory biomarkers in the associations between major chemical pollutants and biomarkers of biological aging, we performed mediation analyses. These analyses used a continuous WQS index as an integrated exposure metric for the chemical mixtures. All models in our study were adjusted for age, sex, BMI, smoking status (current/non-current smoker), alcohol status (current/non-current drinker), TL at baseline, physical activity (yes/no), education level (junior high and below, senior high, college and above), and workplace (coke oven top, coke oven side/bottom, adjunct workplace, office).

To explore the potential mechanisms underlying the effects of urinary metals and PAHs on telomere length, a bioinformatic analysis was performed using the Comparative Toxicogenomics Database (CTD) and GeneCards. In the CTD chemical module, we queried “Se,” “1-OHP,” and “Mo” with the species restricted to *Homo sapiens*. Genes with at least one curated interaction (interaction count ≥ 1) were retained, as described by Wu et al. (23). For GeneCards, we retrieved target genes associated with each chemical and further filtered them by retaining only those with a Relevance score above the median for that chemical (24, 25). This analysis aimed to identify genes associated with both the key exposures within the mixture and the aging outcome. Kyoto Encyclopedia of Genes and Genomes (KEGG) pathways, was subsequently conducted on these overlapping genes. Furthermore, a protein–protein interaction (PPI) network was constructed for the overlapping genes using the STRING database to elucidate potential molecular interactions. Hub genes within this network were identified by applying a high-confidence interaction score threshold of 0.900 (26). All analyses were performed using the R software, version 4.4.5. The level of statistical significance was determined to be *p* < 0.05.

## 3 Results

### 3.1 Baseline characteristics

The study population comprised 867 participants with a mean age of 40.5 ± 7.1 years, 88.6% of whom were male. The majority (90.8%) were non-office coke oven workers, distributed across the following worksites: auxiliary production area (*n* = 543), furnace-side and bottom area (*n* = 132), and furnace top (*n* = 112). Additionally, 60.2% of the participants were current smokers. The urinary concentrations of chemicals among the worker participants were substantially higher than those in the general population. The median levels were approximately 1.5 to 2-fold greater. Detailed overall characteristics are presented in Table 1. The 10 urinary PAHs exhibited median levels ranging from 0.30 μg/mmol creatinine (2-OHPh) to 3.23 μg/mmol creatinine (1-OHP), with ΣOH-PAHs summing to 12.05 μg/mmol creatinine. The 22 metals showed median concentrations spanning 0.016 μg/mmol creatinine (Co) to 187.13 μg/mmol creatinine (Rb).

**Table 1:** Characteristics of the study participants (*N* = 867).

Characteristics	Total
Age, years	40.5 ± 7.1
Male, *n* (%)	768 (88.6)
BMI (kg/m^2^)	23.85 ± 3.14
Current smoking, *n* (%)	522 (60.2)
Current drinking, *n* (%)	307 (35.4)
Exercise, *n* (%)	409 (47.2)
Education level, *n* (%)	
College and above	370 (42.7)
Senior high	351 (40.5)
Junior high and below	146 (16.8)
Workplace, n (%)	
Auxiliary workshop	543 (62.6)
Furnace bottom and side	132 (15.2)
Furnace top	112 (12.9)
Office	80 (9.2)
Al	4.79 (2.45, 8.59)
As	2.82 (1.85, 4.66)
Ba	0.47 (0.26, 0.87)
Cd	0.08 (0.05, 0.12)
Pb	0.43 (0.28, 0.67)
Sb	0.04 (0.02, 0.09)
Sn	1.31 (0.81, 2.15)
Ti	4.68 (2.37, 7.90)
Tl	0.05 (0.03, 0.07)
Cr	0.13 (0.08, 0.23)
Ni	0.18 (0.10, 0.31)
Rb	187.13 (119.93, 298.86)
Sr	11.75 (7.31, 17.93)
V	0.04 (0.02, 0.06)
W	0.02 (0.01, 0.03)
Co	0.016 (0.011, 0.027)
Cu	0.73 (0.50, 1.11)
Fe	8.11 (4.50, 16.20)
Mn	0.27 (0.14, 0.55)
Mo	3.91 (2.47, 6.51)
Se	0.82 (0.53, 1.25)
Zn	30.90 (20.24, 51.16)
1-OHP	3.23(1.87, 5.77)
4-OHPh	0.34 (0.14, 0.71)
9-OHPh	0.74 (0.40, 1.37)
1-OHNa	1.67 (0.97, 3.09)
1-OHPh	0.88 (0.43, 1.60)
2-OHNa	1.65 (0.90, 2.79)
3-OHPh	0.38 (0.20, 0.69)
2-OHPh	0.30 (0.18, 0.55)
2-OHFlu	0.90 (0.58, 1.60)
9-OHFlu	0.61 (0.26, 1.39)
BPDE-Alb adducts in plasma	4.23 (3.63, 4.93)
ΣOH-PAHs	12.05 (8.12, 19.71)

Categorical variables were presented as n (%). Continuous variables were presented as mean ± SD or median (25th, 75th percentiles). The urinary concentrations of 22 metals and 10 PAHs are reported in μg per mmol creatinine, and the plasma concentration of BPDE-Alb adducts is reported in ng per mg albumin.

### 3.2 Multivariable linear regression model

In the single-exposure models, only Mo demonstrated a significant inverse association with the TL-ratio after false discovery rate (FDR) correction for multiple comparisons. When all 33 chemicals were mutually adjusted for in the multi-exposure model, significant associations were observed between the TL-ratio and several urinary metals and PAH metabolites (Mo, Se, Tl, Ti, As, and 1-OHP; all *p* < 0.05). Among these, Se exhibited the largest effect size (*β* = −0.256, 95% CI: −0.492 to −0.021). Specifically, Mo, Se, and 1-OHP were significantly negatively associated with the TL-ratio (Table 2). Regarding baseline mtDNAcn, urinary Ba was the only analyte showing a significant positive association in both the single- and multi-exposure models. These results are detailed in Supplementary Table S1.

**Table 2:** Associations of urinary PAHs and metals with aging biomarkers.

Exposed chemicals	Multiple-exposure models^a^			
	TL-ratio		mtDNAcn	
	β (95% CI)	*P* value	β (95% CI)	*P* value
Mo	−0.185 (−0.323, −0.046)	**0.009**	0.002 (−0.107, 0.112)	0.966
Se	−0.256 (−0.492, −0.021)	**0.033**	0.008 (−0.177, 0.194)	0.931
Rb	−0.120 (−0.382, 0.143)	0.372	0.046 (−0.161, 0.253)	0.665
Cr	−0.069 (−0.204, 0.066)	0.317	0.101 (−0.005, 0.208)	0.062
W	−0.029 (−0.094, 0.037)	0.393	0.021 (−0.030, 0.073)	0.422
TI	0.165 (0.033, 0.297)	**0.015**	−0.063 (−0.254, 0.130)	0.522
Cu	−0.034 (−0.207, 0.139)	0.698	0.163 (0.027, 0.298)	**0.019**
Co	−0.042 (−0.201, 0.117)	0.601	−0.046 (−0.172, 0.079)	0.469
Pb	−0.012 (−0.151, 0.126)	0.862	−0.006 (−0.116, 0.103)	0.914
Sn	0.026 (−0.137, 0.189)	0.757	−0.033 (−0.161, 0.096)	0.618
Zn	0.053 (−0.108, 0.213)	0.520	−0.023 (−0.150, 0.103)	0.720
Al	0.063 (−0.212, 0.085)	0.403	−0.017 (−0.134, 0.100)	0.772
Sr	−0.023 (−0.181, 0.135)	0.772	0.051 (−0.176, 0.073)	0.418
Sb	−0.007 (−0.103, 0.088)	0.884	0.038 (−0.038, 0.113)	0.326
Ni	−0.000 (−0.079, 0.078)	0.989	−0.001 (−0.079, 0.078)	0.989
Mn	−0.051 (−0.214, 0.111)	0.537	−0.028 (−0.157, 0.100)	0.665
Cd	0.141 (−0.026, 0.307)	0.099	−0.081 (−0.213, 0.050)	0.225
Ba	0.046 (−0.072, 0.165)	0.441	0.154 (0.061, 0.247)	**0.001**
As	0.182 (0.026, 0.339)	**0.022**	−0.015 (−0.139, 0.108)	0.807
Fe	0.111 (−0.013, 0.235)	0.079	−0.078 (−0.176, 0.019)	0.116
V	0.026 (−0.028, 0.080)	0.343	−0.001 (−0.044, 0.041)	0.946
Ti	0.165 (0.033, 0.297)	**0.015**	0.014 (−0.118, 0.145)	0.798
1-OHP	−0.166 (−0.307, −0.026)	**0.020**	0.035 (−0.037, 0.106)	0.342
4-OHPh	−0.028 (−0.067, 0.011)	0.164	0.002 (−0.029, 0.034)	0.904
9-OHPh	−0.033 (−0.134, 0.068)	0.523	0.046 (−0.034, 0.125)	0.260
1-OHNa	−0.101 (−0.280, 0.077)	0.267	−0.037 (−0.178, 0.104)	0.604
1-OHPh	0.032 (−0.059, 0.123)	0.492	0.035 (−0.037, 0.106)	0.342
2-OHNa	0.117 (−0.054, 0.289)	0.180	0.049 (−0.086, 0.185)	0.478
3-OHPh	−0.040 (−0.126, 0.045)	0.358	0.033 (−0.034, 0.101)	0.333
2-OHPh	0.119 (−0.002, 0.236)	**0.047**	0.035 (−0.127, 0.057)	0.461
2-OHFlu	0.054 (−0.058, 0.165)	0.345	0.036 (−0.052, 0.124)	0.426
9-OHFlu	−0.006 (−0.049, 0.037)	0.780	0.008 (−0.026, 0.042)	0.656
BPDE-Alb adducts in plasma	0.030 (−0.121, 0.182)	0.696	−0.017 (−0.136, 0.102)	0.777

The urinary concentrations of 22 metals (μg/mmol creatine) and 10 PAHs (μg/mmol creatine), and plasma level of BPDE-Alb adducts (ng/mg albumin), were ln-transformed in the regression models.

a In the multiple-exposure models, all chemicals were included simultaneously. The medels was adjusted for age, sex, BMI, smoking status (current/non-current smoker), alcohol status (current/non-current drinker), TL at baseline, physical activity (yes/no), education level (junior high and below, senior high, college and above), and workplace (coke oven top, coke oven side/bottom, adjunct workplace, office). Bold values indicate statistical significance at P < 0.05.

### 3.3 Weighted quantile sum regression model

**Figure 1:**
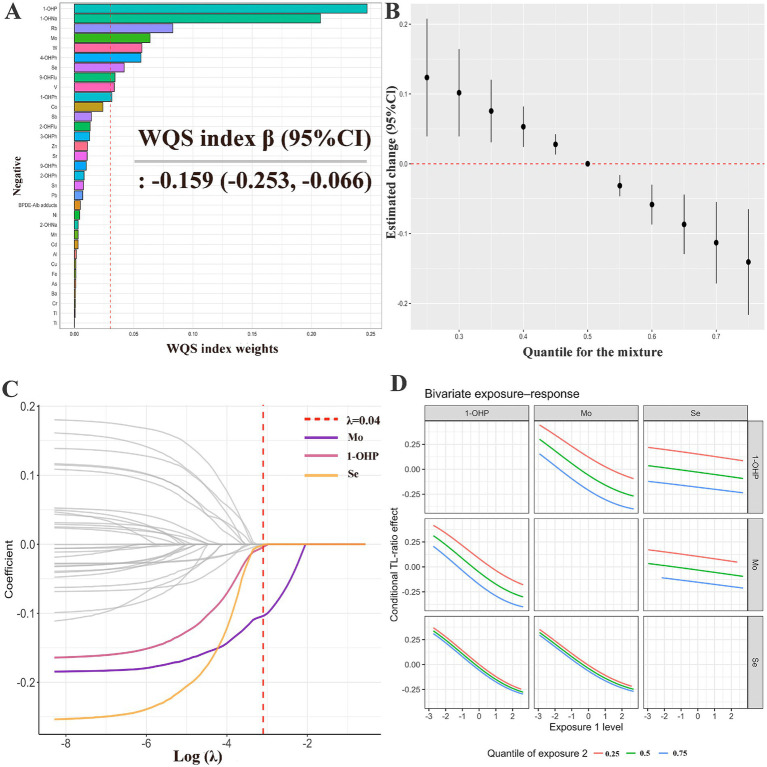
Associations of the metals and PAHs mixture with the TL-ratio. **(A)** WQS regression model of the 33-chemical mixture and the TL-ratio; **(B)** Overall effect of the mixture on the TL-ratio across exposure percentiles relative to the 50th percentile, estimated by the BKMR model; **(C)** LASSO regression coefficient profile for the associations between exposures and the TL-ratio; **(D)** Bivariate exposure-response functions showing the relationship between one exposure and the TL ratio at different fixed percentiles (25th, 50th, 75th) of a second exposure, with all other exposures held at their medians. All models were adjusted for age, sex, body mass index (BMI), smoking status (current/non-current smoker), alcohol consumption (current/non-current drinker), baseline TL, physical activity (yes/no), education level (junior high and below, senior high, college and above), and workplace (coke oven top, coke oven side/bottom, adjunct workplace, office).

After adjusting for all covariates, the WQS regression analysis revealed a statistically significant adverse composite effect of co-exposure to PAHs and multiple metals on TL-ratio (*β* = −0.159, 95% CI: −0.253, −0.066) (Figure 1A). In the WQS model constrained to a negative effect direction, the highest weights among the individual chemicals were assigned to 1-OHP, 1-OHNa, Rb, and Mo. No significant positive composite effect was identified (Supplementary Figure S5). Furthermore, the chemical mixture exposure showed a statistically significant positive composite association with baseline mtDNAcn in the WQS model (*β* = 0.123, 95% CI: 0.047, 0.200) (Supplementary Figure S6).

### 3.4 BKMR model and LASSO penalized regression analyses

Based on the hierarchical BKMR model, the association between the mixture of urinary PAH metabolites and metals and aging biomarkers was further evaluated. Results from the BKMR analysis were consistent with the WQS findings, indicating an inverse association between the chemical mixture and TL-ratio across its entire concentration range, which was statistically significant (Figure 1B). Additionally, a positive overall association was observed between the mixture and mtDNAcn in the BKMR model (Supplementary Figure S7). Detailed results of the BKMR model are presented in Supplementary Figures S8–S11.

The relative contribution of specific chemicals to the overall mixture effect was assessed using the conditional posterior inclusion probability (CondPIP) (Supplementary Table S2). For TL-ratio, Mo (CondPIP: 0.701) and 1-OHP (CondPIP: 0.872) showed the highest CondPI*p* values within their respective groups, each of which had a group posterior inclusion probability (GroupPIP) meeting the threshold of 0.5. Ba exhibited the highest CondPIP value for mtDNAcn. Furthermore, dimensionality reduction was performed on the 33 chemicals using the LASSO regression (Supplementary Figure S12). The optimal model fit, indicated by the minimum mean squared error (MSE), was achieved at a *λ* value of 0.04. At this penalty, Mo, Se, and 1-OHP were identified as significant predictors in the TL-ratio model (Figure 1C).

Finally, BKMR analysis found no interaction among the three significant chemicals (1-OHP, Se, and Mo). This was evidenced by their bivariate exposure-response functions, which showed parallel curves with identical slopes when the other exposures were fixed at their 25th, 50th, and 75th percentiles (Figure 1D).

### 3.5 Restricted cubic spline models

**Figure 2:**
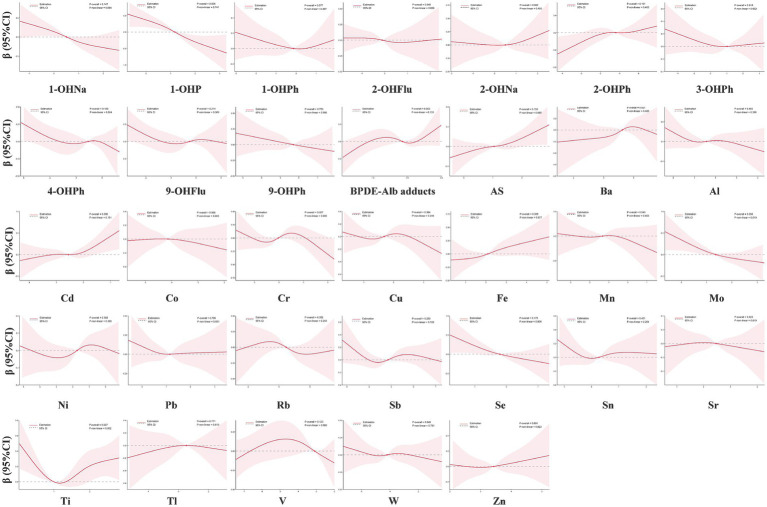
The dose–response associations of urinary PAHs and metals with TL-ratio based on RCS regression model. Knots were placed at the 10th, 50th, and 90th percentiles of pollutants, and the reference value was set at the 50th percentile. Models were adjusted for age, sex, BMI, smoking status (current/non-current smoker), alcohol status (current/non-current drinker), TL at baseline, physical activity (yes/no), education level (junior high and below, senior high, college and above), and workplace (coke oven top, coke oven side/bottom, adjunct workplace, office). Solid lines indicate *β*, and shadow shape indicate 95% CIs.

Using RCS models, we explored potential non-linear dose–response relationships between each exposure and the outcomes, adjusting for all other chemicals in the model (Figure 2). The results indicated a negative, linear dose–response relationship between 1-OHP and TL-ratio (*P* for overall association < 0.05; *P* for non-linearity > 0.05). A similar negative linear trend was observed for Mo. Although not statistically significant, Mo and Se exhibited an approximate L-shaped non-linear association with TL-ratio. In contrast, a positive and non-linear dose–response relationship was observed between Ba and mtDNAcn (*P* for overall association < 0.001; *P* for non-linearity < 0.001). This relationship showed a steep increase at lower concentrations of Ba, which attenuated and plateaued at higher concentrations (Supplementary Figure S13).

### 3.6 Mediation analysis of oxidative stress and inflammation

**Figure 3:**
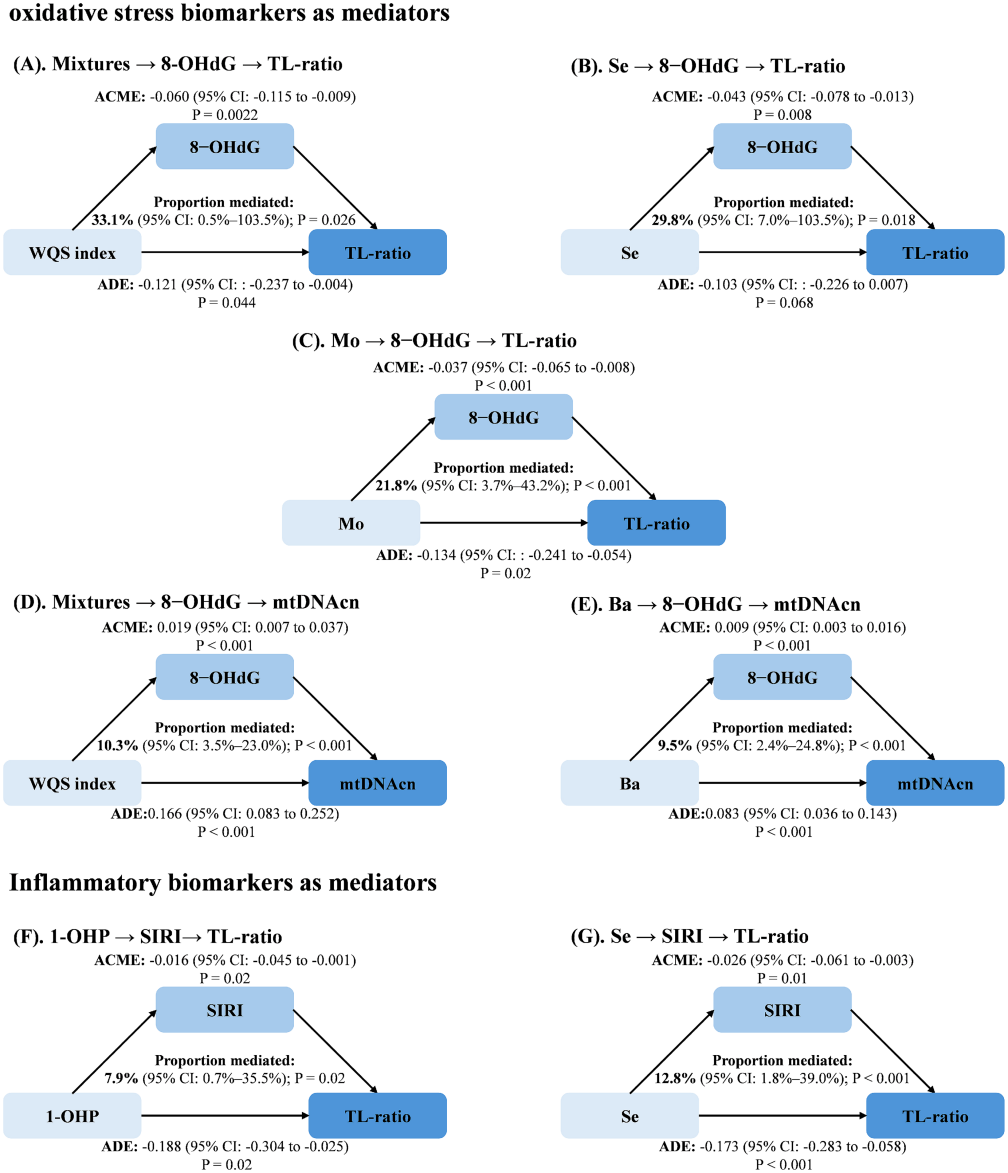
Mediating effects of mediators on the associations between PAHs and metals and aging biomarkers in the study population. **(A–C)** Mediating effects of 8-OHdG on the associations of the chemical mixture, Se, and Mo with TL-ratio, respectively. **(D, E)** Mediating effects of 8-OHdG on the associations of the chemical mixture and Ba with mtDNAcn, respectively. **(F, G)** Mediating effects of SIRI on the associations of 1-OHP and Se with TL-ratio, respectively. Models were adjusted for age, sex, BMI, smoking status (current/non-current smoker), alcohol status (current/non-current drinker), TL at baseline, physical activity (yes/no), education level (junior high and below, senior high, college and above), and workplace (coke oven top, coke oven side/bottom, adjunct workplace, office).

The mediating effects of oxidative stress and inflammation on the associations between 33 chemical mixture exposure and aging biomarkers were investigated, as summarized in Figure 3. Overall, 8-OHdG mediated 33.1% of the association between the chemical mixture (represented by the WQS index) and TL-ratio. Specifically, it accounted for 29.8 and 21.8% of the associations of Se and Mo with TL-ratio, respectively. 8-OHdG also significantly mediated 10.3% of the association between mixture and mtDNAcn. For the SIRI, it mediated 12.8 and 7.9% of the associations of Se and 1-OHP with TL-ratio, respectively. Detailed results of the mediation analysis are provided in Supplementary Table S3.

### 3.7 Network toxicology analysis

**Figure 4:**
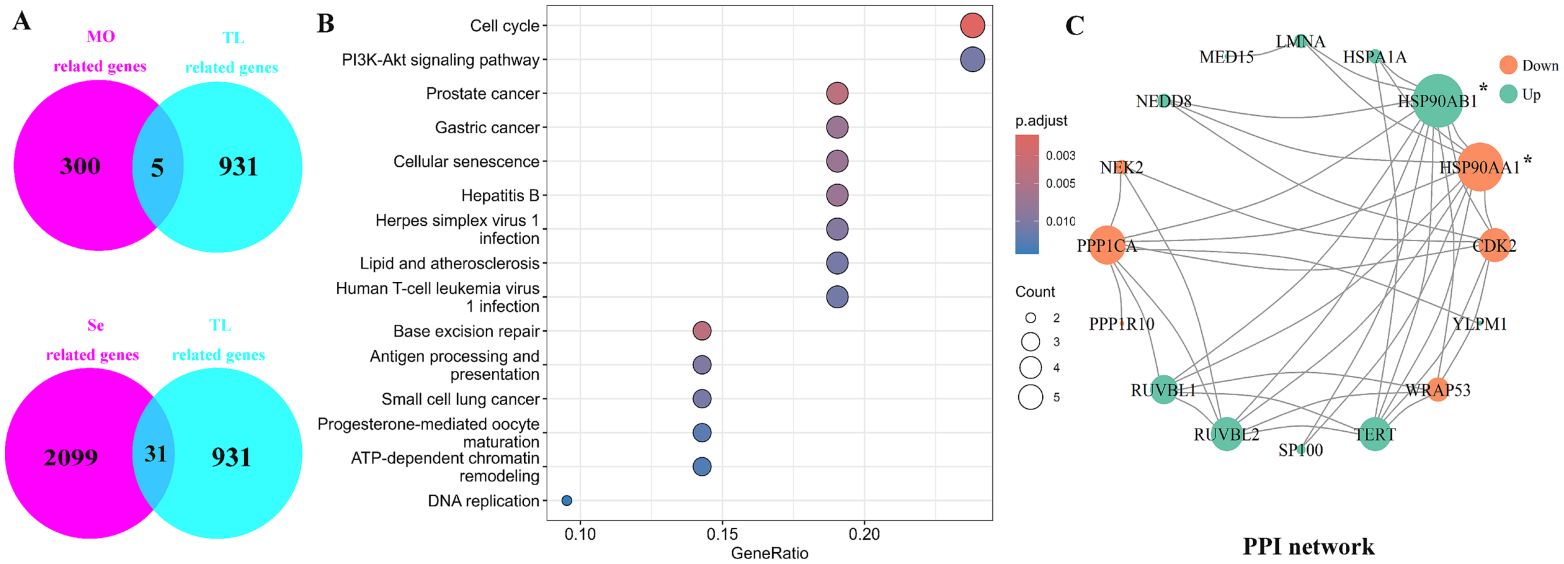
Network toxicology analysis framework. **(A)** Workflow and results for screening overlapping target genes between the identified exposures and telomere length regulation. **(B)** Enriched KEGG pathways of the overlapping genes. **(C)** Protein–protein interaction (PPI) network of the overlapping genes.

The CTD and GeneCards databases were queried to identify genes associated with the three key chemicals linked to telomere length. After combining the results from both databases and applying the relevance score filter for GeneCards, we identified 88, 300, and 2,099 associated genes for 1-OHP, Mo, and Se, respectively (Figure 4A). These gene sets were then subjected to an overlap analysis with the 931 genes associated with telomere length regulation. The results indicated 1 shared gene for 1-OHP, 5 shared genes for Mo, and 31 shared genes for Se. The detailed lists of overlapping genes for each chemical are provided in Supplementary Table S4. Due to the limited number of analyzable genes for Mo and 1-OHP, they were excluded from subsequent analyses. To explore the biological mechanisms and key targets among the shared genes, KEGG pathway enrichment and PPI network analyses were performed on the 31 genes common to Se and telomere length. KEGG enrichment analysis highlighted “Cell cycle” and “PI3K-Akt signaling pathway” as key pathways within the Cellular Processes and Environmental Information Processing categories, respectively (Figure 4B). Furthermore, PPI network analysis identified *HSP90AA1* and *HSP90AB1* as core target genes linking Se exposure to telomere length regulation (Figure 4C).

## 4 Discussion

Unlike the relevant recent literature (27–29), this is the first prospective cohort study in an occupational population to quantify the combined effect of simultaneous exposure to metals and PAHs on aging biomarkers using WQS and BKMR models, and to explore their potential interactions. Both the WQS and BKMR models in our study consistently indicated that elevated urinary concentrations of metals and PAHs were significantly associated with telomere attrition and mtDNAcn. Furthermore, by integrating mediation analysis with the mixture effects identified by the WQS model, we observed that 8-OHdG mediated 33.1% of the association between mixed metal and PAH exposure and telomere attrition.

Numerous previous studies have reported significant negative associations between individual exposure to either metals or PAHs and telomere length, which is largely consistent with our main findings (13, 14, 30). In a cross-sectional study of coke oven workers similar to our population, Manuela et al. reported shorter TL in exposed workers compared to controls, while mtDNAcn levels were similar (31). Furthermore, a recent study of 6,478 participants from the Shenzhen Aging-Related Disorders Cohort measured urinary concentrations of 22 trace metals and found a positive and statistically significant association between joint metal exposure and elevated mtDNAcn (32). Mitochondrial DNA not only represents long-term accumulated damage but also reflects dynamic mitochondrial stress responses and compensatory mitochondrial biosynthesis. An increase in the mtDNAcn may indicate a compensatory response of mitochondria to environmental stress. Another study observed that Ba deficiency, leading to decreased mtDNAcn, was linked to an increased risk of type 2 diabetes during aging (33). These recent findings align with ours, further suggesting an important association between co-exposure to high levels of metals and PAHs and altered biomarkers of biological aging. Studies on oxidative stress and inflammation in relation to urinary metals and PAHs are also consistent with our results, highlighting their key role in DNA damage processes (14, 34–36). Pollutant-enhanced, reactive oxygen species-induced oxidative stress serves as a primary molecular initiating event, driving biological inflammation and toxicity, and ultimately accelerating biological aging.

In contrast to other studies, our investigation specifically focused on workers employed for over 1 year in a coke oven plant, a population with substantially higher exposure levels to metals and PAHs compared to the general population (16, 37). Numerous previous studies, including population-based and murine experiments, have reported that excessively high urinary concentrations of metals such as Se and Mo can disrupt redox homeostasis, induce oxidative stress, and subsequently lead to testicular DNA damage and apoptosis (38–40). One population-based study also conducted in Wuhan demonstrated significant inverse correlations between urinary Mo and Se levels and sperm quality, with Mo remaining significant in fully adjusted models (41). Notably, the median urinary Se concentration in that study was similar to ours (11.2 μg/L) and 1.45 times higher than that reported for a community-based population in Wuhan (7.7 μg/L) (42). It should be noted that our exposure assessment for Se and Mo relied solely on urinary concentrations, which may have limitations. Selenium homeostasis is tightly regulated by the body; urinary excretion primarily reflects recent dietary intake or excess above requirements rather than long-term body burden (43, 44). In contrast, molybdenum has a relatively short biological half-life and is excreted predominantly via urine, making urinary concentrations a more reliable indicator of short-term exposure.

The chaperone mechanism of Heat Shock Protein 90 (HSP90) is a key regulator of proteostasis in eukaryotic cells under both physiological and stress conditions. It is involved in numerous cellular processes beyond protein folding, including DNA repair, development, immune response, and neurodegenerative diseases (45). Our comprehensive bioinformatic analysis revealed that Se may participate in telomere regulation through multiple molecular pathways, with down regulation of *HSP90AA1* and upregulation of *HSP90AB1* identified as a core process. *HSP90AA1* and *HSP90AB1* regulate oxidative stress and telomerase activity by modulating the synthesis of HSP90, which facilitates and stabilizes the PI3K-Akt signaling pathway (46–48). While these findings, together with ours, suggest that both deficiency and excess of Se may pose health risks, the mechanistic pathways proposed by our mediation and network toxicology analyses remain exploratory and must be interpreted with caution.

The main strengths of our study include its longitudinal cohort design among coke oven workers, providing the first comprehensive assessment of the combined effects of metals and PAHs on aging biomarkers in an occupational population typically co-exposed to high levels of these pollutants, thereby offering a more holistic understanding of environmental exposure risks. Secondly, the application of both WQS and BKMR models allowed for mutual validation and complementarity in assessing the chemical mixture, enhancing the robustness of our findings. Finally, we explored potential mechanisms underlying the associations of both the pollutant mixture and individual pollutants with aging biomarkers, including mediation analysis and network toxicology. We also acknowledge several important limitations. Although urinary metals are widely used in occupational and environmental epidemiology, they primarily reflect recent exposure and may not fully capture cumulative or tissue-specific accumulation, especially for metals with longer biological half-lives (e.g., cadmium, lead). Future studies incorporating multiple biomatrices (e.g., blood, hair, toenails) are needed to validate and complement urinary measurements (49). Second, the generalizability of our findings is limited as the participants were coke oven workers and the sample size was relatively restricted due to stringent inclusion and exclusion criteria. Future studies should include more diverse populations to strengthen the evidence. Furthermore, the study sample was predominantly male (88.6%), which limits the generalizability of our findings to female occupational populations. Finally, network toxicology is a hypothesis-generating tool; it identifies potential targets for future investigation but cannot establish causal mechanisms without experimental validation. Further *in vivo* and *in vitro* studies are needed to confirm the specific biological pathways proposed here. Nonetheless, the results and insights from this longitudinal cohort study remain informative and relevant for this specific occupational group. Although we adjusted for important covariates such as basic demographics and specific work environments, we could not account for all potential confounders. Finally, due to limitations in blood sample availability, we could not validate the mtDNAcn analysis longitudinally.

## 5 Conclusion

In summary, this study demonstrates that co-exposure to PAHs and metals is associated with telomere attrition and elevated mtDNAcn. The oxidative stress biomarker 8-OHdG and the novel inflammation index SIRI mediated the associations of individual pollutants (1-OHP, Mo, Se) and the 33-chemical mixture with aging biomarkers. HSP90 was identified as a core molecular target linking Se exposure to TL regulation through network toxicology analysis. These findings provide a theoretical foundation for understanding the integrated risks of human exposure to complex environmental pollutants.

## Data Availability

The datasets presented in this article are not readily available because data will be made available on request. Requests to access the datasets should be directed to Xuefeng Lai, xuefeng_lai416@wust.edu.cn.
