# Supplementary material for: Associations of co-exposure to metals and polycyclic aromatic hydrocarbons with aging biomarkers: insights from epidemiology and network toxicology
Source: Front Public Health. 2026 May 28;14:1844457. doi: 10.3389/fpubh.2026.1844457 (PMC13253628; doi:10.3389/fpubh.2026.1844457)
Supplement: Supplementary file 1 [file Supplementary_file_1.docx]

**Supplementary materials**

**Table S1.** Associations of urinary PAHs and metals concentrations with aging biomarkers in single and multiple exposure linear regression models.

**Table S2.** The PIPs of BKMR models.

**Table S3.** Mediated effect of SIRI on the association of urinary PAHs and metals concentrations with aging biomarkers.

**Table S4.** Network toxicology results for Se, Mo and 1‑OHP.

**Fig. S1.** Flow chart of participants included in the study.

**Fig. S2.** Pearson correlations among ln-transformed pollutant concentrations.

**Fig. S3.** Distribution of telomere length (2010–2014) and its change.

**Fig. S4.** (A) Elbow plot for optimal exposure-group number; (B) Hierarchical clustering assignment.

**Fig. S5.** Association of TL-ratio with co-exposure to metals and PAHs mixtures by WQS (positive) analyses.

**Fig. S6.** Association of mtDNAcn with co-exposure to metals and PAHs mixtures by WQS (positive: A, negative: B) analyses.

**Fig. S7.** Joint effect of the metal and PAH mixture on mtDNAcn estimated by the BKMR model.

**Fig. S8.** Associations of individual metals and PAHs with TL-ratio from the BKMR model, with all other mixture components fixed at their 25th (red), 50th (green), or 75th (blue) percentiles.

**Fig. S9.** Associations of individual metals and PAHs with mtDNAcn from the BKMR model, with all other mixture components fixed at their 25th (red), 50th (green), or 75th (blue) percentiles.

**Fig. S10.** Univariate exposure-response relationships between mixture and TL-ratio in the BKMR model.

**Fig. S11.** Univariate exposure-response relationships between mixture and mtDNAcn in the BKMR model.

**Fig. S12.**The LASSO penalized regression analysis for the associations between mixture and aging biomarkers.

**Fig. S13.**The dose-response association of urinary PAHs and metals with mtDNAcn based on RCS regression model.

**Table S1.** Associations of urinary PAHs and metals concentrations with aging biomarkers in single and multiple exposure linear regression models (N=867).

| Exposed  chemicals | | **TL-ratio** | | | | | |  | **mtDNAcn** | | | | | |
| --- | --- | --- | --- | --- | --- | --- | --- | --- | --- | --- | --- | --- | --- | --- |
|  |  | Single-exposure models^a^ | | |  | Multiple-exposure models | |  | Single-exposure models | |  | Multiple-exposure models | |  |
|  |  | β (95% CI) | *P* value | |  | β (95% CI) | *P* value |  | β (95% CI) | *P* value |  | β (95% CI) | *P* value |  |
| **Metals in urine** | | | | | | | | | | | | | | |
| Mo | -0.158 (-0.245, -0.071) | | | **0.013^b^** |  | -0.185 (-0.323, -0.046) | 0.009 |  | -0.009 (-0.081, 0.063) | 0.99 |  | 0.002 (-0.107, 0.112) | 0.966 |  |
| Se | -0.144 (-0.244, -0.044) | | | 0.084 |  | -0.256 (-0.492, -0.021) | 0.033 |  | 0.005 (-0.075, 0.085) | 0.99 |  | 0.008 (-0.177, 0.194) | 0.931 |  |
| Rb | -0.120 (-0.218, -0.022) | | | 0.171 |  | -0.120 (-0.382, 0.143) | 0.372 |  | -0.023 (-0.099, 0.053) | 0.99 |  | 0.046 (-0.161, 0.253) | 0.665 |  |
| Cr | -0.085 (-0.164, -0.006) | | | 0.218 |  | -0.069 (-0.204, 0.066) | 0.317 |  | 0.059 (-0.004, 0.122) | 0.648 |  | 0.101 (-0.005, 0.208) | 0.062 |  |
| W | -0.059 (-0.115, -0.003) | | | 0.218 |  | -0.029 (-0.094, 0.037) | 0.393 |  | 0.037 (-0.016, 0.090) | 0.648 |  | 0.021 (-0.030, 0.073) | 0.422 |  |
| TI | -0.100 (-0.198, -0.002) | | | 0.218 |  | 0.165 (0.033, 0.297) | 0.015 |  | -0.009 (-0.087, 0.069) | 0.99 |  | -0.063 (-0.254, 0.130) | 0.522 |  |
| Cu | -0.096 (-0.193, 0.001) | | | 0.218 |  | -0.034 (-0.207, 0.139) | 0.698 |  | 0.080 (0.003, 0.157) | 0.648 |  | 0.163 (0.027, 0.298) | 0.019 |  |
| Co | -0.084 (-0.172, 0.004) | | | 0.218 |  | -0.042 (-0.201, 0.117) | 0.601 |  | -0.009 (-0.078, 0.060) | 0.99 |  | -0.046 (-0.172, 0.079) | 0.469 |  |
| Pb | -0.069 (-0.154, 0.016) | | | 0.312 |  | -0.012 (-0.151, 0.126) | 0.862 |  | 0.036 (-0.032, 0.104) | 0.856 |  | -0.006 (-0.116, 0.103) | 0.914 |  |
| Sn | -0.066 (-0.151, 0.019) | | | 0.312 |  | 0.026 (-0.137, 0.189) | 0.757 |  | 0.008 (-0.058, 0.074) | 0.99 |  | -0.033 (-0.161, 0.096) | 0.618 |  |
| Zn | -0.073 (-0.167, 0.021) | | | 0.312 |  | 0.053 (-0.108, 0.213) | 0.52 |  | 0.008 (-0.067, 0.082) | 0.99 |  | -0.023 (-0.150, 0.103) | 0.72 |  |
| Al | -0.052 (-0.120, 0.016) | | | 0.312 |  | 0.063 (-0.212, 0.085) | 0.403 |  | 0.026 (-0.028, 0.080) | 0.856 |  | -0.017 (-0.134, 0.100) | 0.772 |  |
| Sr | -0.066 (-0.158, 0.026) | | | 0.345 |  | -0.023 (-0.181, 0.135) | 0.772 |  | 0.006 (-0.067, 0.079) | 0.99 |  | 0.051 (-0.176, 0.073) | 0.418 |  |
| Sb | -0.045 (-0.112, 0.022) | | | 0.393 |  | -0.007 (-0.103, 0.088) | 0.884 |  | 0.038 (-0.015, 0.091) | 0.648 |  | 0.038 (-0.038, 0.113) | 0.326 |  |
| Ni | -0.035 (-0.091, 0.021) | | | 0.424 |  | -0.000 (-0.079, 0.078) | 0.989 |  | -0.022 (-0.066, 0.022) | 0.856 |  | -0.001 (-0.079, 0.078) | 0.989 |  |
| Mn | -0.034 (-0.096, 0.028) | | | 0.505 |  | -0.051 (-0.214, 0.111) | 0.537 |  | 0.044 (-0.006, 0.094) | 0.648 |  | -0.028 (-0.157, 0.100) | 0.665 |  |
| Cd | -0.043 (-0.135, 0.049) | | | 0.552 |  | 0.141 (-0.026, 0.307) | 0.099 |  | 0.000 (-0.073, 0.074) | 0.99 |  | -0.081 (-0.213, 0.050) | 0.225 |  |
| Ba | -0.022 (-0.081, 0.037) | | | 0.667 |  | 0.046 (-0.072, 0.165) | 0.441 |  | 0.081 (0.035, 0.128) | **0.023^b^** |  | 0.154 (0.061, 0.247) | 0.001 |  |
| As | -0.031 (-0.127, 0.065) | | | 0.69 |  | 0.182 (0.026, 0.339) | 0.022 |  | -0.021 (-0.097, 0.055) | 0.99 |  | -0.015 (-0.139, 0.108) | 0.807 |  |
| Fe | -0.016 (-0.081, 0.049) | | | 0.759 |  | 0.111 (-0.013, 0.235) | 0.079 |  | -0.014 (-0.066, 0.038) | 0.99 |  | -0.078 (-0.176, 0.019) | 0.116 |  |
| V | -0.008 (-0.048, 0.030) | | | 0.759 |  | 0.026 (-0.028, 0.080) | 0.343 |  | -0.008 (-0.039, 0.023) | 0.99 |  | -0.001 (-0.044, 0.041) | 0.946 |  |
| Ti | -0.038 (-0.114, 0.038) | | | 0.529 |  | 0.165 (0.033, 0.297) | 0.015 |  | -0.002 (-0.062, 0.058) | 0.99 |  | 0.014 (-0.118, 0.145) | 0.798 |  |
| **PAH metabolites in urine** | | | | | | | | | | | | | | |
| 1-OHP | -0.090 (-0.169, -0.011) | | | 0.202 |  | -0.166 (-0.307, -0.026) | 0.02 |  | -0.044 (-0.106, 0.018) | 0.648 |  | 0.035 (-0.037, 0.106) | 0.342 |  |
| 4-OHPh | -0.033 (-0.070, 0.004) | | | 0.252 |  | -0.028 (-0.067, 0.011) | 0.164 |  | 0.004 (-0.026, 0.034) | 0.99 |  | 0.002 (-0.029, 0.034) | 0.904 |  |
| 9-OHPh | -0.033 (-0.098, 0.032) | | | 0.529 |  | -0.033 (-0.134, 0.068) | 0.523 |  | 0.016 (-0.036, 0.068) | 0.99 |  | 0.046 (-0.034, 0.125) | 0.26 |  |
| 1-OHNa | -0.047 (-0.125, 0.031) | | | 0.448 |  | -0.101 (-0.280, 0.077) | 0.267 |  | 0.009 (-0.052, 0.070) | 0.99 |  | -0.037 (-0.178, 0.104) | 0.604 |  |
| 1-OHPh | -0.019 (-0.080, 0.042) | | | 0.702 |  | 0.032 (-0.059, 0.123) | 0.492 |  | -0.004 (-0.053, 0.045) | 0.99 |  | 0.035 (-0.037, 0.106) | 0.342 |  |
| 2-OHNa | -0.012 (-0.096, 0.072) | | | 0.85 |  | 0.117 (-0.054, 0.289) | 0.18 |  | 0.016 (-0.050, 0.082) | 0.99 |  | 0.049 (-0.086, 0.185) | 0.478 |  |
| 3-OHPh | -0.005 (-0.064, 0.054) | | | 0.922 |  | -0.040 (-0.126, 0.045) | 0.358 |  | 0.025 (-0.022, 0.072) | 0.856 |  | 0.033 (-0.034, 0.101) | 0.333 |  |
| 2-OHPh | 0.004 (-0.062, 0.070) | | | 0.936 |  | 0.119 (-0.002, 0.236) | 0.047 |  | 0.000 (-0.064, 0.064) | 0.99 |  | 0.035 (-0.127, 0.057) | 0.461 |  |
| 2-OHFlu | 0.001 (-0.071, 0.073) | | | 0.972 |  | 0.054 (-0.058, 0.165) | 0.345 |  | 0.042 (-0.015, 0.099) | 0.648 |  | 0.036 (-0.052, 0.124) | 0.426 |  |
| 9-OHFlu | -0.013 (-0.050, 0.024) | | | 0.667 |  | -0.006 (-0.049, 0.037) | 0.78 |  | 0.020 (-0.008, 0.048) | 0.648 |  | 0.008 (-0.026, 0.042) | 0.656 |  |
| BPDE-Alb adducts in plasma | -0.033 (-0.180, 0.114) | | | 0.759 |  | 0.030 (-0.121, 0.182) | 0.696 |  | 0.005 (-0.112, 0.122) | 0.99 |  | -0.017 (-0.136, 0.102) | 0.777 |  |

**Note:** The urinary concentrations of 22 metals (μg/mmol creatine) and 10 PAHs (μg/mmol creatine), and plasma level of BPDE-Alb adducts (ng/mg albumin), were ln-transformed in the regression models.

^a^ In the single-exposure models, each chemical level was analyzed separately, while in the multiple-exposure models, all chemicals were included simultaneously. Both models were adjusted for age, sex, BMI, smoking status (current/non-current smoker), alcohol status (current/non-current drinker), TL at baseline, physical activity (yes/no), education level (junior high and below, senior high, college and above), and workplace (coke oven top, coke oven side/bottom, adjunct workplace, office).

^b^ Significant associations after false discovery rate (FDR) < 0.05.

**Table S2.** The PIPs of BKMR models.

| Exposed chemicals | | **TL-ratio** | | | |  | **mtDNAcn** | | |
| --- | --- | --- | --- | --- | --- | --- | --- | --- | --- |
|  |  | group | groupPIP | | condPIP |  | group | groupPIP | condPIP |
| Mo | 1 | | | 0.6760 | 0.7006 |  | 1 | 1.000 | 1.000 |
| Se | 1 | | | 0.6760 | 0.0547 |  | 1 | 1.000 | 1.000 |
| Rb | 1 | | | 0.6760 | 0.0577 |  | 1 | 1.000 | 1.000 |
| Cr | 1 | | | 0.6760 | 0.0112 |  | 1 | 1.000 | 1.000 |
| W | 1 | | | 0.6760 | 0.0275 |  | 1 | 1.000 | 1.000 |
| TI | 1 | | | 0.6760 | 0.0166 |  | 1 | 1.000 | 1.000 |
| Cu | 1 | | | 0.6760 | 0.008 |  | 1 | 1.000 | 1.000 |
| Co | 1 | | | 0.6760 | 0.0207 |  | 1 | 1.000 | 1.000 |
| Pb | 1 | | | 0.6760 | 0.0118 |  | 1 | 1.000 | 1.000 |
| Sn | 1 | | | 0.6760 | 0.0216 |  | 1 | 1.000 | 1.000 |
| Zn | 1 | | | 0.6760 | 0.0056 |  | 1 | 1.000 | 1.000 |
| Al | 1 | | | 0.6760 | 0.0086 |  | 1 | 1.000 | 1.000 |
| Sr | 1 | | | 0.6760 | 0.0118 |  | 1 | 1.000 | 1.000 |
| Sb | 1 | | | 0.6760 | 0.0018 |  | 1 | 1.000 | 1.000 |
| Ni | 2 | | | 0.4066 | 0.5047 |  | 2 | 0.2436 | 0.4064 |
| Mn | 1 | | | 0.6760 | 0.0062 |  | 1 | 1.000 | 1.000 |
| Cd | 1 | | | 0.6760 | 0.0107 |  | 1 | 1.000 | 1.000 |
| Ba | 1 | | | 0.6760 | 0.0027 |  | 1 | 1.000 | 1.000 |
| As | 1 | | | 0.6760 | 0.0044 |  | 1 | 1.000 | 1.000 |
| Fe | 1 | | | 0.6760 | 0.003 |  | 1 | 1.000 | 1.000 |
| V | 2 | | | 0.4066 | 0.4953 |  | 2 | 0.2436 | 0.5936 |
| Ti | 1 | | | 0.6760 | 0.0148 |  | 1 | 1.000 | 1.000 |
| 1-OHP | 3 | | | 0.6894 | 0.8721 |  | 3 | 0.3506 | 0.1164 |
| 4-OHPh | 4 | | | 0.5592 | 0.2135 |  | 4 | 0.3610 | 0.1352 |
| 9-OHPh | 3 | | | 0.6894 | 0.0102 |  | 3 | 0.3506 | 0.4062 |
| 1-OHNa | 3 | | | 0.6894 | 0.0189 |  | 3 | 0.3506 | 0.1004 |
| 1-OHPh | 3 | | | 0.6894 | 0.016 |  | 3 | 0.3506 | 0.0188 |
| 2-OHNa | 3 | | | 0.6894 | 0.0279 |  | 3 | 0.3506 | 0.1101 |
| 3-OHPh | 3 | | | 0.6894 | 0.0087 |  | 3 | 0.3506 | 0.1329 |
| 2-OHPh | 3 | | | 0.6894 | 0.0267 |  | 3 | 0.3506 | 0.0251 |
| 2-OHFlu | 3 | | | 0.6894 | 0.0197 |  | 3 | 0.3506 | 0.0901 |
| 9-OHFlu | 4 | | | 0.5592 | 0.3251 |  | 4 | 0.3610 | 0.2432 |
| BPDE-Alb adducts in plasma | 4 | | | 0.5592 | 0.4614 |  | 4 | 0.3610 | 0.6216 |

**Note:** The urinary concentrations of 22 metals (μg/mmol creatine) and 10 PAHs (μg/mmol creatine), and plasma level of BPDE-Alb adducts (ng/mg albumin), were ln-transformed in the regression models. Both models were adjusted for age, sex, BMI, smoking status (current/non-current smoker), alcohol status (current/non-current drinker), TL at baseline, physical activity (yes/no), education level (junior high and below, senior high, college and above), and workplace (coke oven top, coke oven side/bottom, adjunct workplace, office).

**Table S3.** Mediated effect of SIRI on the association of urinary PAHs and metals concentrations with aging biomarkers.

|  | | | **Direct effect** | ***P* value** | | **Indirect effect** | ***P* value** | | | **Proportion mediated, %** | ***P* value** |  |  |
| --- | --- | --- | --- | --- | --- | --- | --- | --- | --- | --- | --- | --- | --- |
| **SIRI** | | | | | | | | | | | | |  |
| **TL-ratio** | | | | | | | | | | | | |  |
| Mo | -0.1913 (-0.3473, -0.0374) | | | 0.02 | 0.0057 (-0.0063, 0.0192) | | | 0.28 | -0.0307 (-0.3639, 0.0335) | | 0.30 | | |
| WQS index | -0.1876 (-0.3041, -0.0254) | | | <0.001 | -0.0160 (-0.0453, -0.0013) | | | 0.7 | 0.0787 (0.0067, 0.3547) | | 0.7 | | |
| **mtDNAcn** | | | | | | | | | | | | | |
| Ba | | 0.1441 (0.0445, 0.2625) | | 0.04 | -0.0044 (-0.0272, 0.0077) | | | 0.48 | -0.0313 (-0.3837, 0.0705) | | 0.48 | | |
| WQS index | | 0.1018 (0.0344, 0.1698) | | 0.04 | -0.0048 (-0.0176, 0.0025) | | | 0.24 | -0.0493 (-0.3324, 0.0363) | | 0.26 | | |

**Note:** The model was adjusted for age, sex, BMI, smoking status (current/non-current smoker), alcohol status (current/non-current drinker), TL at baseline, physical activity (yes/no), education level (junior high and below, senior high, college and above), and workplace (coke oven top, coke oven side/bottom, adjunct workplace, office).

**Table S3.** Mediated effect of SIRI on the association of urinary PAHs and metals concentrations with aging biomarkers.

|  | | | **Direct effect** | ***P* value** | | **Indirect effect** | ***P* value** | | | **Proportion mediated, %** | ***P* value** |  |  |
| --- | --- | --- | --- | --- | --- | --- | --- | --- | --- | --- | --- | --- | --- |
| **SIRI** | | | | | | | | | | | | |  |
| **TL-ratio** | | | | | | | | | | | | |  |
| Mo | -0.1913 (-0.3473, -0.0374) | | | 0.02 | 0.0057 (-0.0063, 0.0192) | | | 0.28 | -0.0307 (-0.3639, 0.0335) | | 0.30 | | |
| WQS index | -0.1876 (-0.3041, -0.0254) | | | <0.001 | -0.0160 (-0.0453, -0.0013) | | | 0.7 | 0.0787 (0.0067, 0.3547) | | 0.7 | | |
| **mtDNAcn** | | | | | | | | | | | | | |
| Ba | | 0.1441 (0.0445, 0.2625) | | 0.04 | -0.0044 (-0.0272, 0.0077) | | | 0.48 | -0.0313 (-0.3837, 0.0705) | | 0.48 | | |
| WQS index | | 0.1018 (0.0344, 0.1698) | | 0.04 | -0.0048 (-0.0176, 0.0025) | | | 0.24 | -0.0493 (-0.3324, 0.0363) | | 0.26 | | |

**Note:** The model was adjusted for age, sex, BMI, smoking status (current/non-current smoker), alcohol status (current/non-current drinker), TL at baseline, physical activity (yes/no), education level (junior high and below, senior high, college and above), and workplace (coke oven top, coke oven side/bottom, adjunct workplace, office).

**Table S4.** Network toxicology results for Se, Mo and 1‑OHP.

| Chemical | | | CTD genes | GeneCards genes (score > median) | | Union genes | Overlap with telomere genes | | | Shared gene symbols |
| --- | --- | --- | --- | --- | --- | --- | --- | --- | --- | --- |
| Se | 2006 | | | 677 | 2099 | | | 31 | HSP90AA1, HSP90AB1, … | |
| Mo | 125 | | | 184 | 300 | | | 5 | MAPK1, MAPK3, PCNA, TP53, HSP90AA1 | |
| 1‑OHP | | 55 | | 44 | 88 | | | 1 | XRCC1 | |

**Note:** CTD, Comparative Toxicogenomics Database; GeneCards genes were filtered by Relevance score > median for each chemical (Se median = 1.35, Mo median = 0.82, 1‑OHP median = 0.46). Union genes represent the combination of CTD‑derived and GeneCards‑derived genes after removal of duplicates.

**
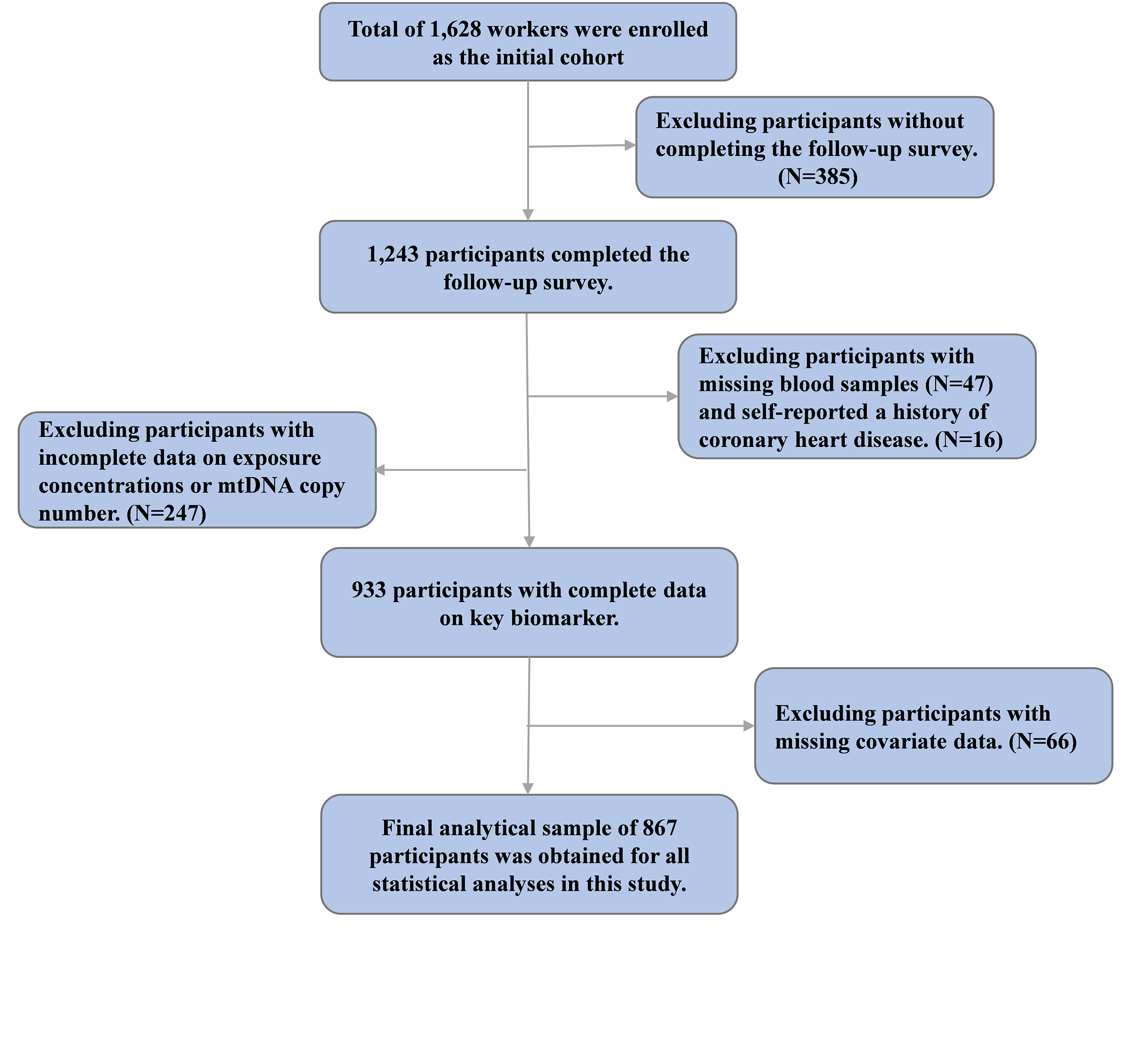
**

**Fig. S1.** Flow chart of participants included in the study.


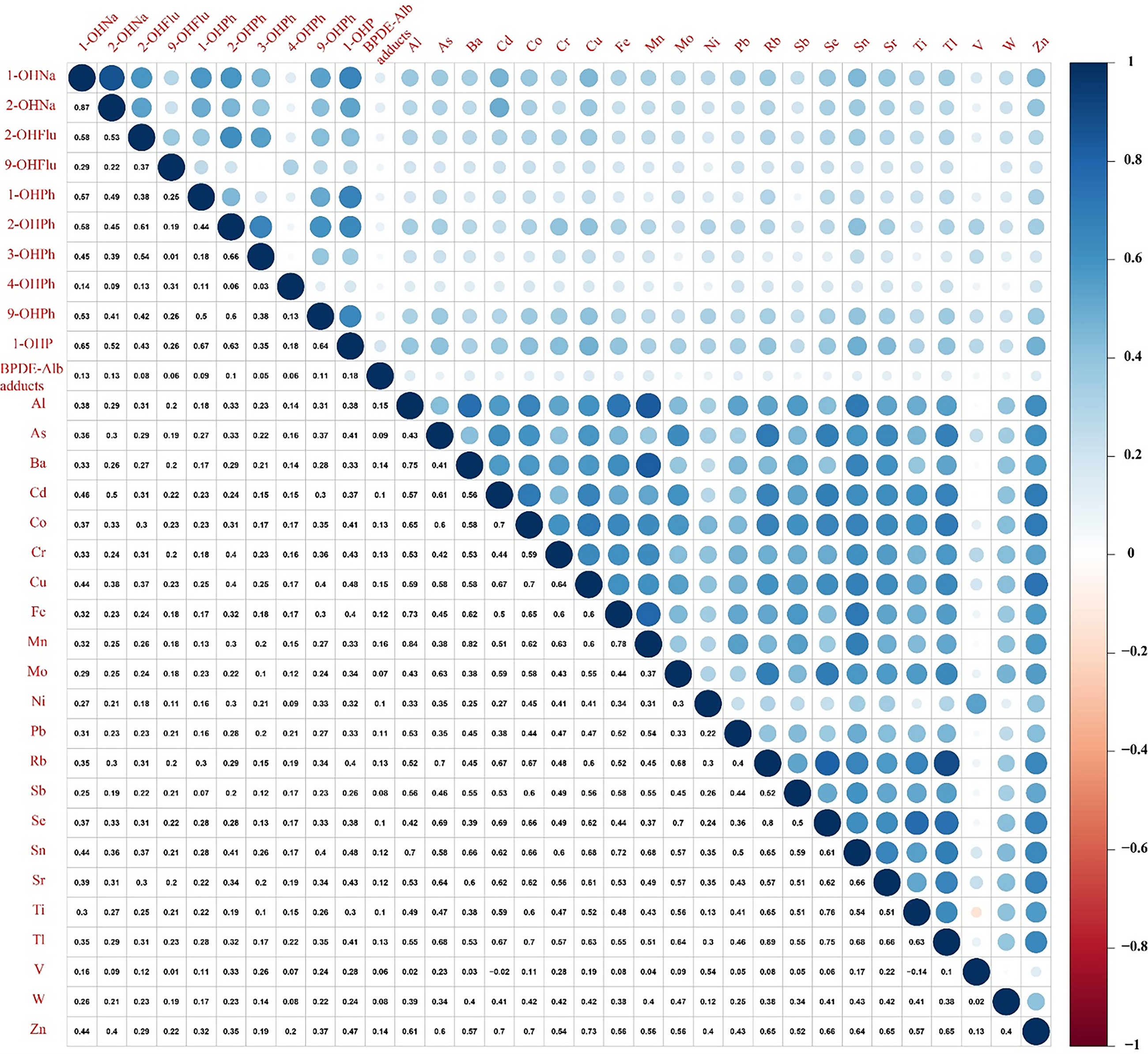


**Fig. S2.** Pearson correlations among ln-transformed pollutant concentrations.

**
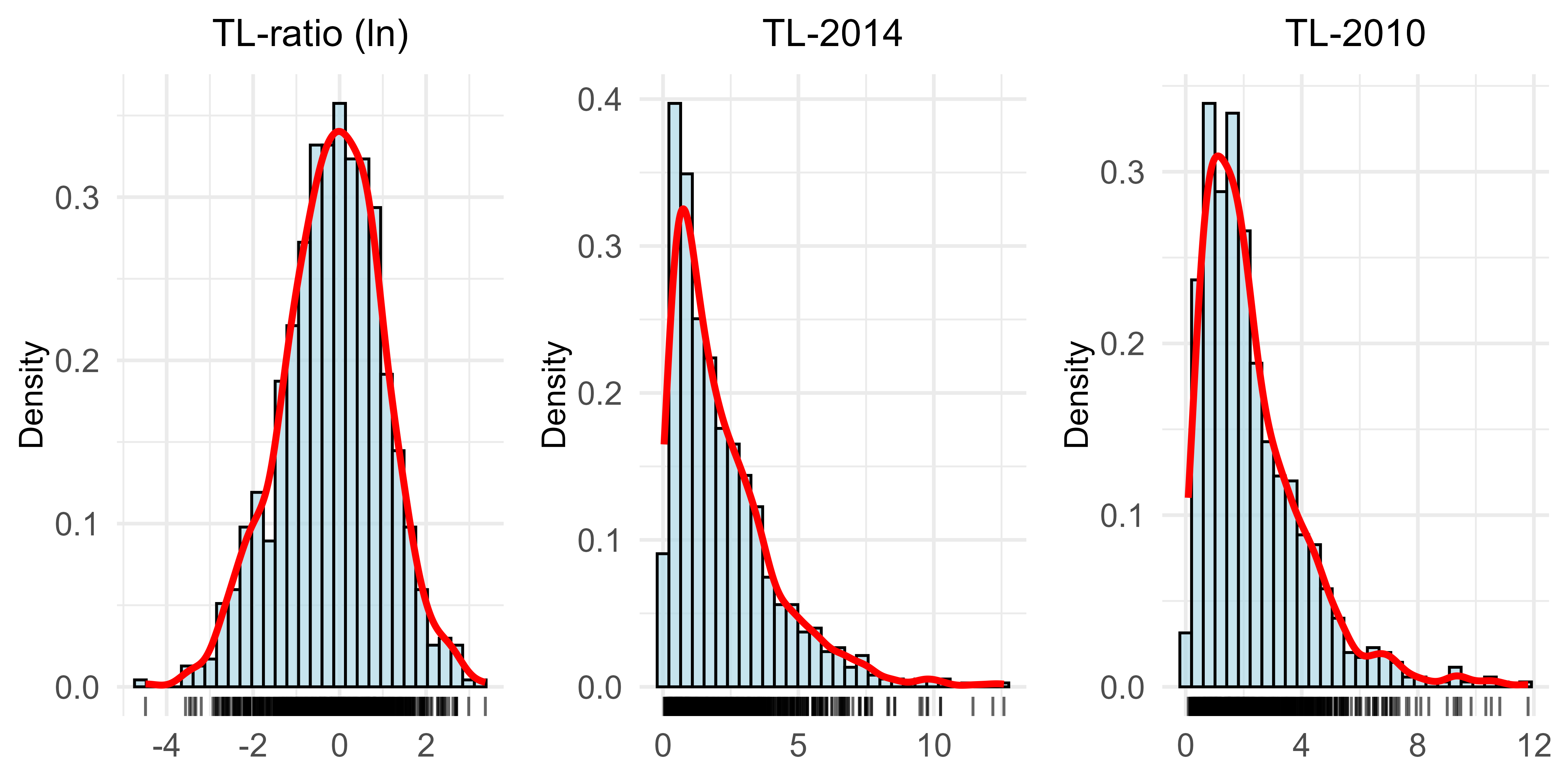
Fig. S3.** Distribution of telomere length (2010–2014) and its change.


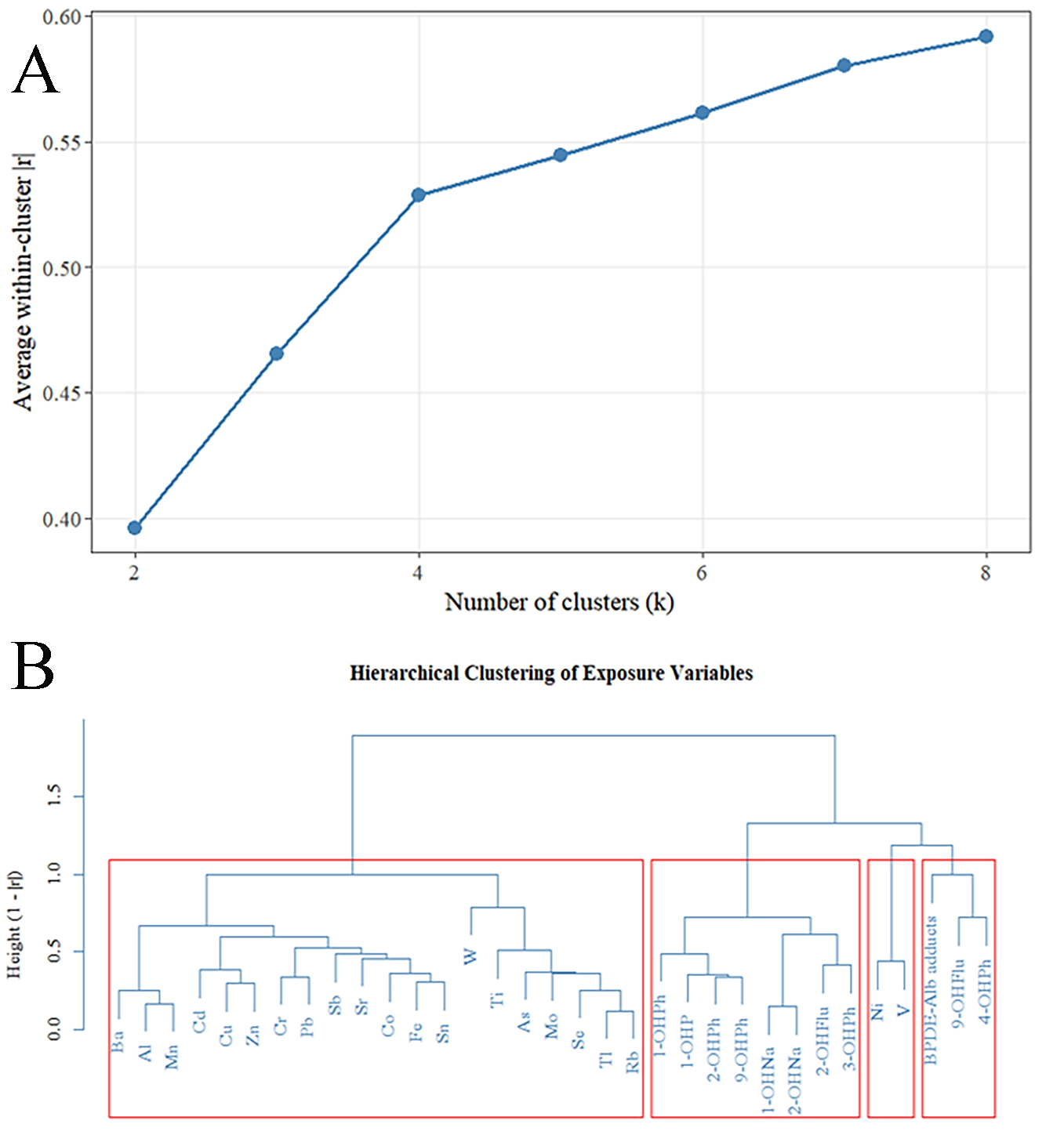


**Fig. S4.** (A) Elbow plot for optimal exposure-group number; (B) Hierarchical clustering assignment.


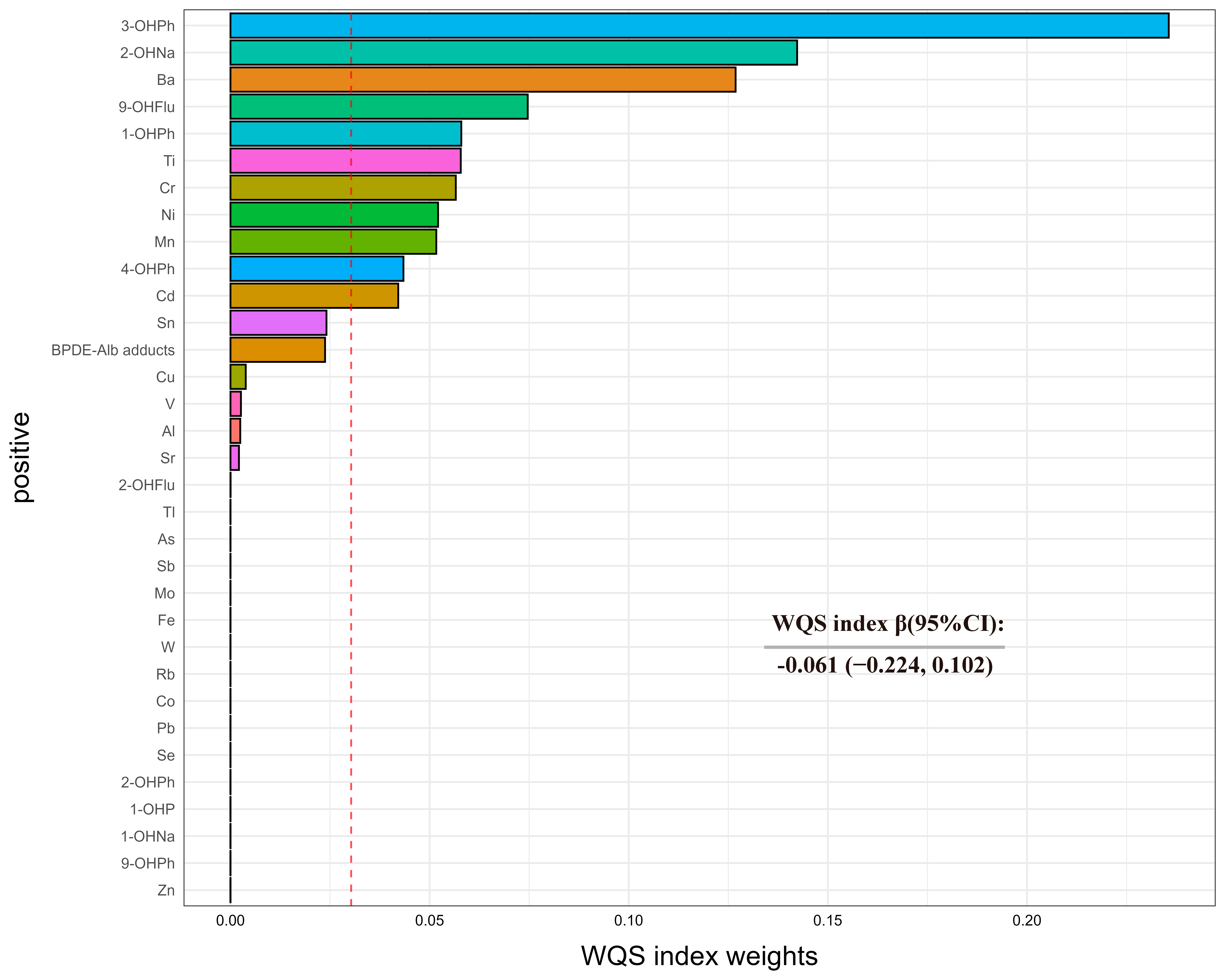


**Fig. S5.** Association of TL-ratio with co-exposure to metals and PAHs mixtures by WQS (positive) analyses. The model was adjusted for age, sex, BMI, smoking status (current/non-current smoker), alcohol status (current/non-current drinker), TL at baseline, physical activity (yes/no), education level (junior high and below, senior high, college and above), and workplace (coke oven top, coke oven side/bottom, adjunct workplace, office).


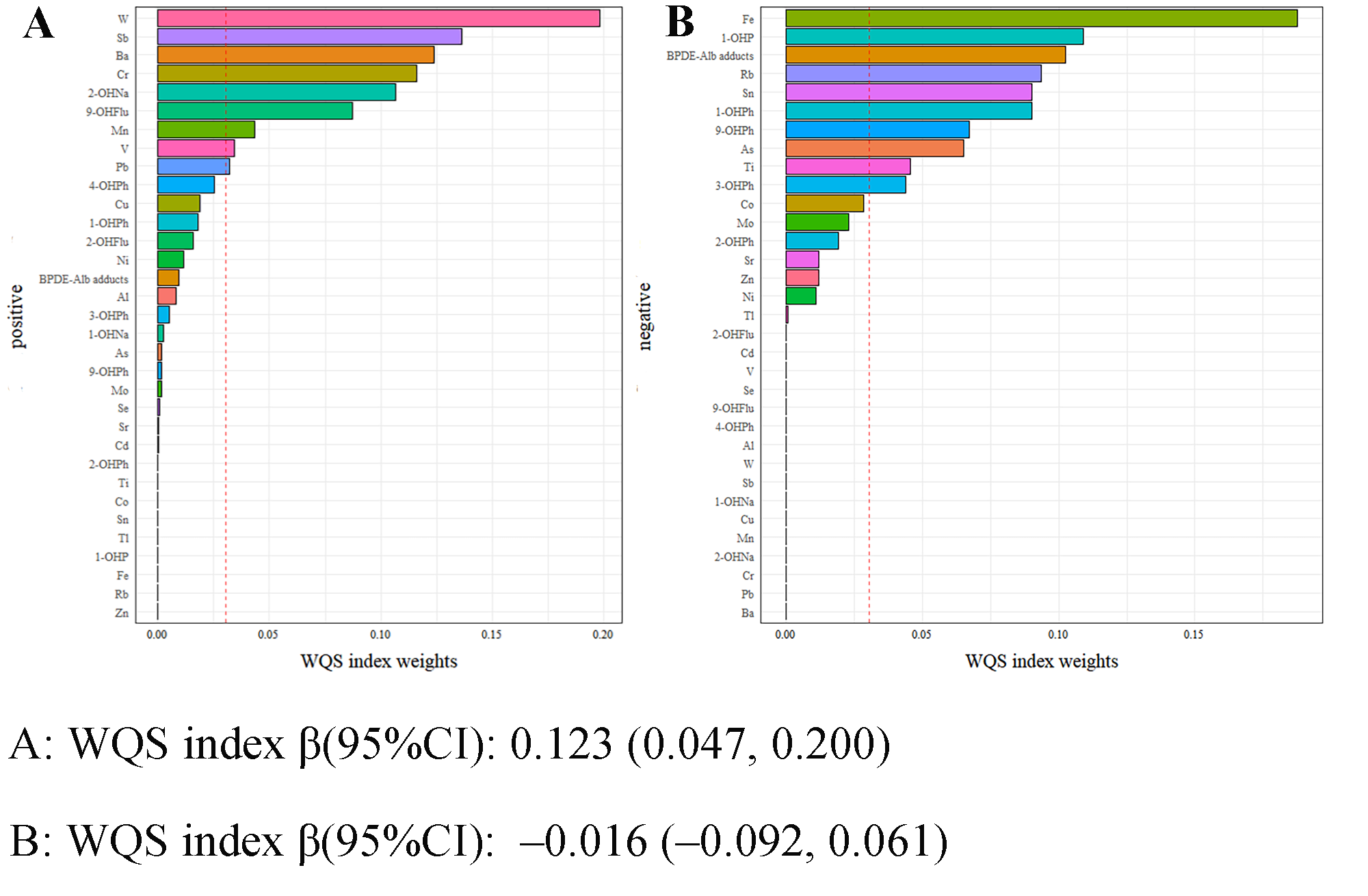


**Fig. S6.** Association of mtDNAcn with co-exposure to metals and PAHs mixtures by WQS (positive: A, negative: B) analyses. The model was adjusted for age, sex, BMI, smoking status (current/non-current smoker), alcohol status (current/non-current drinker), TL at baseline, physical activity (yes/no), education level (junior high and below, senior high, college and above), and workplace (coke oven top, coke oven side/bottom, adjunct workplace, office).


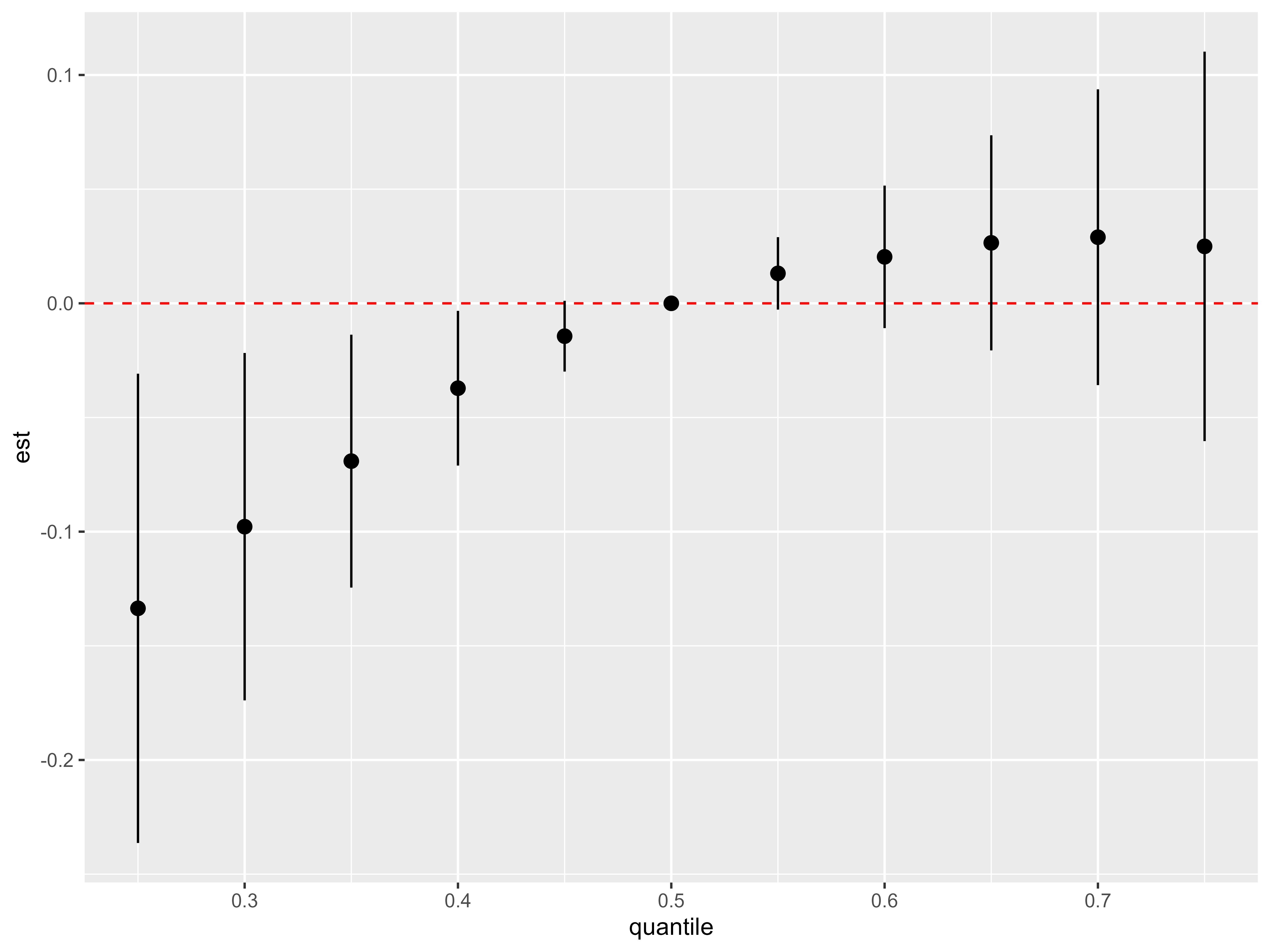


**Fig. S7.** Joint effect of the metal and PAH mixture on mtDNAcn estimated by the BKMR model. The model was adjusted for age, sex, BMI, smoking status (current/non-current smoker), alcohol status (current/non-current drinker), TL at baseline, physical activity (yes/no), education level (junior high and below, senior high, college and above), and workplace (coke oven top, coke oven side/bottom, adjunct workplace, office).


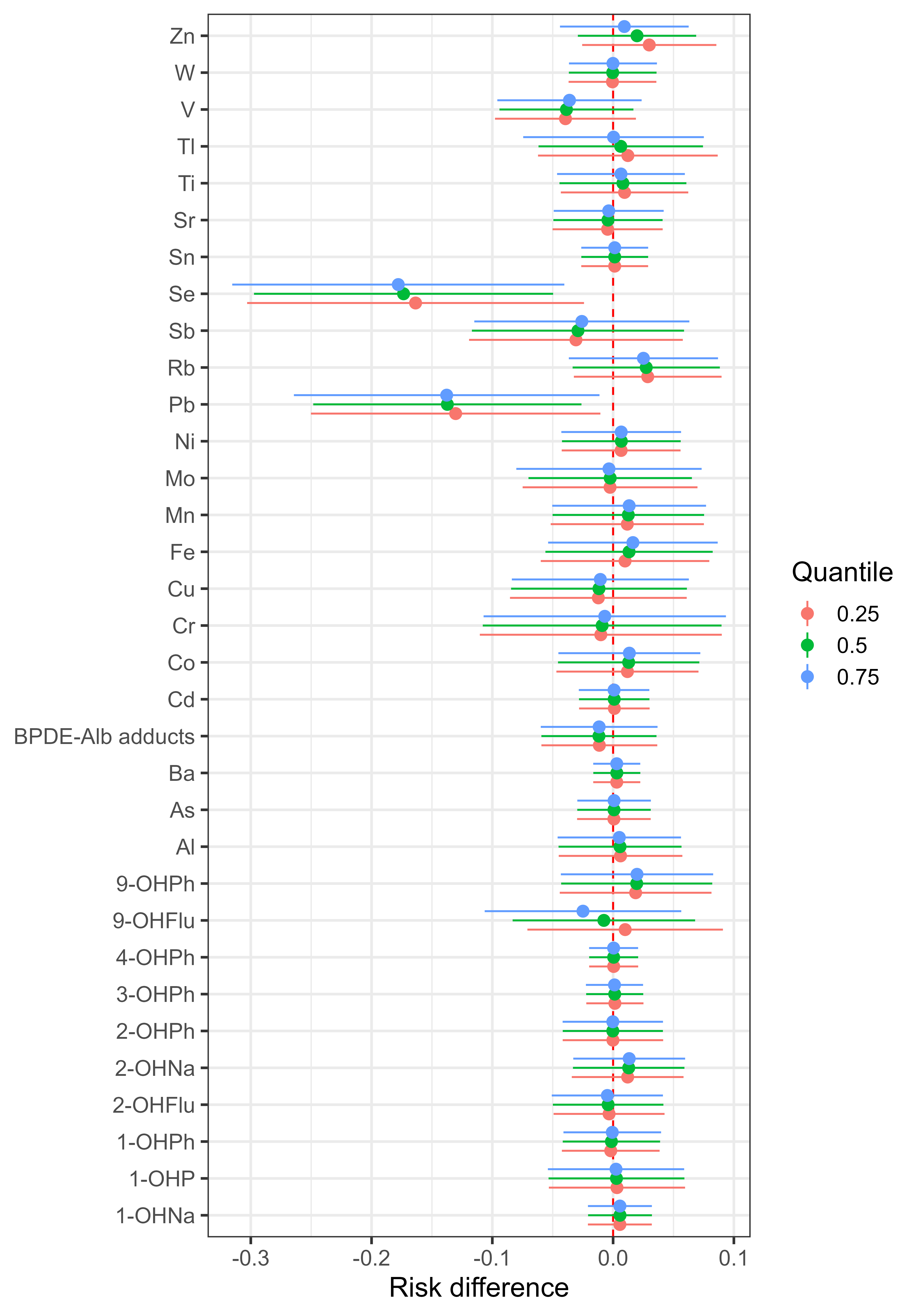


**Fig. S8.** Associations of individual metals and PAHs with TL-ratio from the BKMR model, with all other mixture components fixed at their 25th (red), 50th (green), or 75th (blue) percentiles.


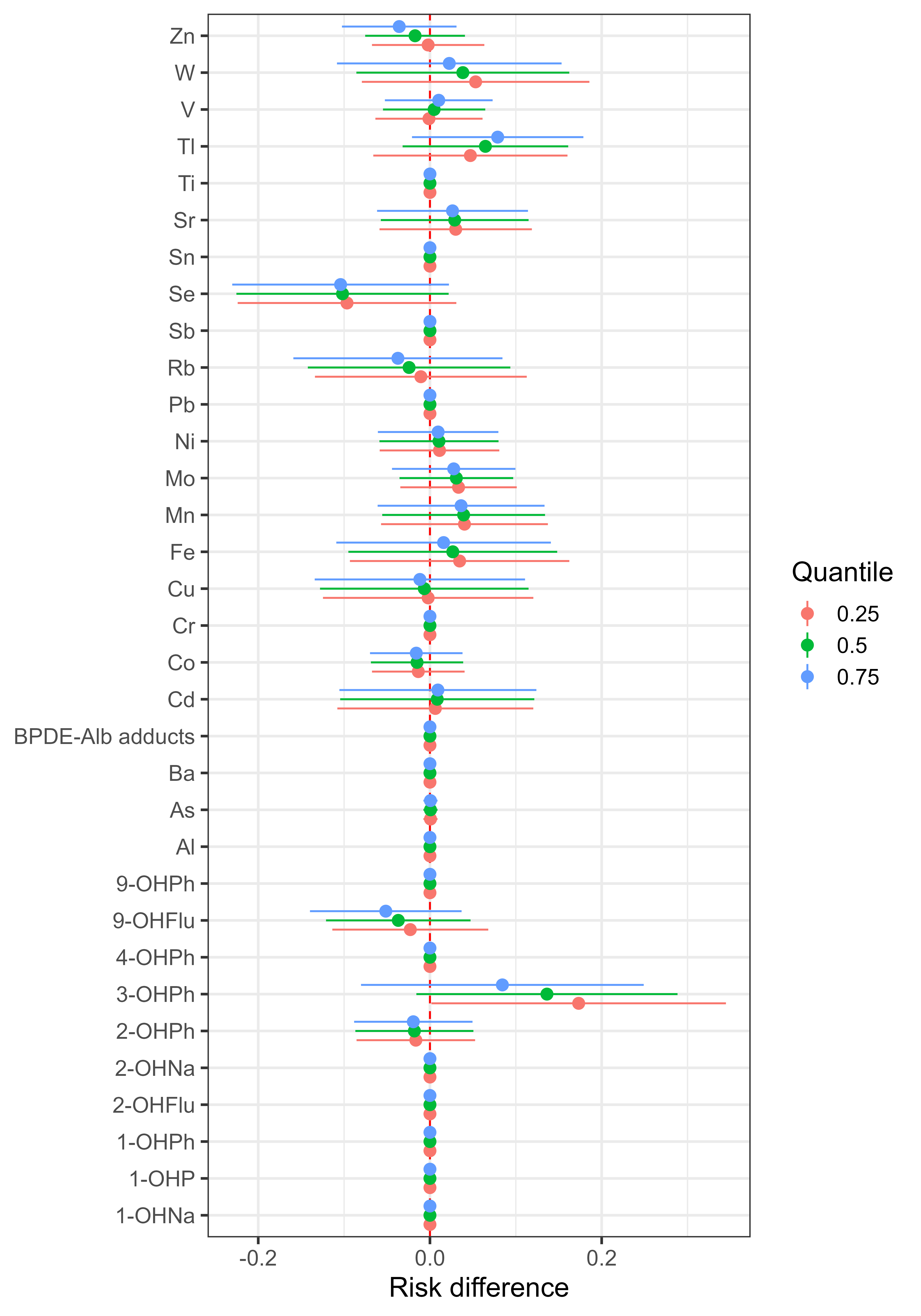


**Fig. S9.** Associations of individual metals and PAHs with mtDNAcn from the BKMR model, with all other mixture components fixed at their 25th (red), 50th (green), or 75th (blue) percentiles.

**
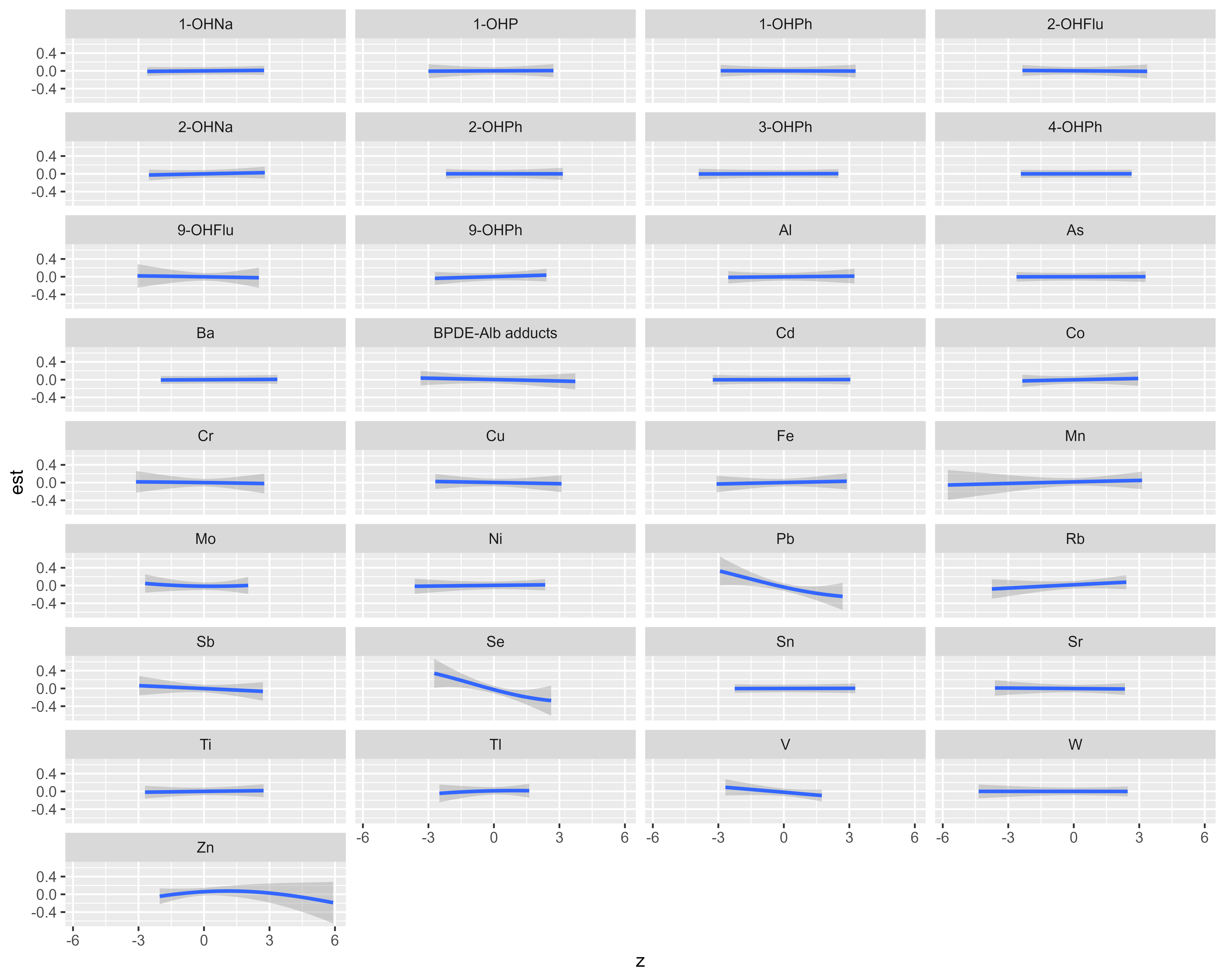
**

**Fig. S10.** Univariate exposure-response relationships between mixture and TL-ratio in the BKMR model. The model was adjusted for age, sex, BMI, smoking status (current/non-current smoker), alcohol status (current/non-current drinker), TL at baseline, physical activity (yes/no), education level (junior high and below, senior high, college and above), and workplace (coke oven top, coke oven side/bottom, adjunct workplace, office).


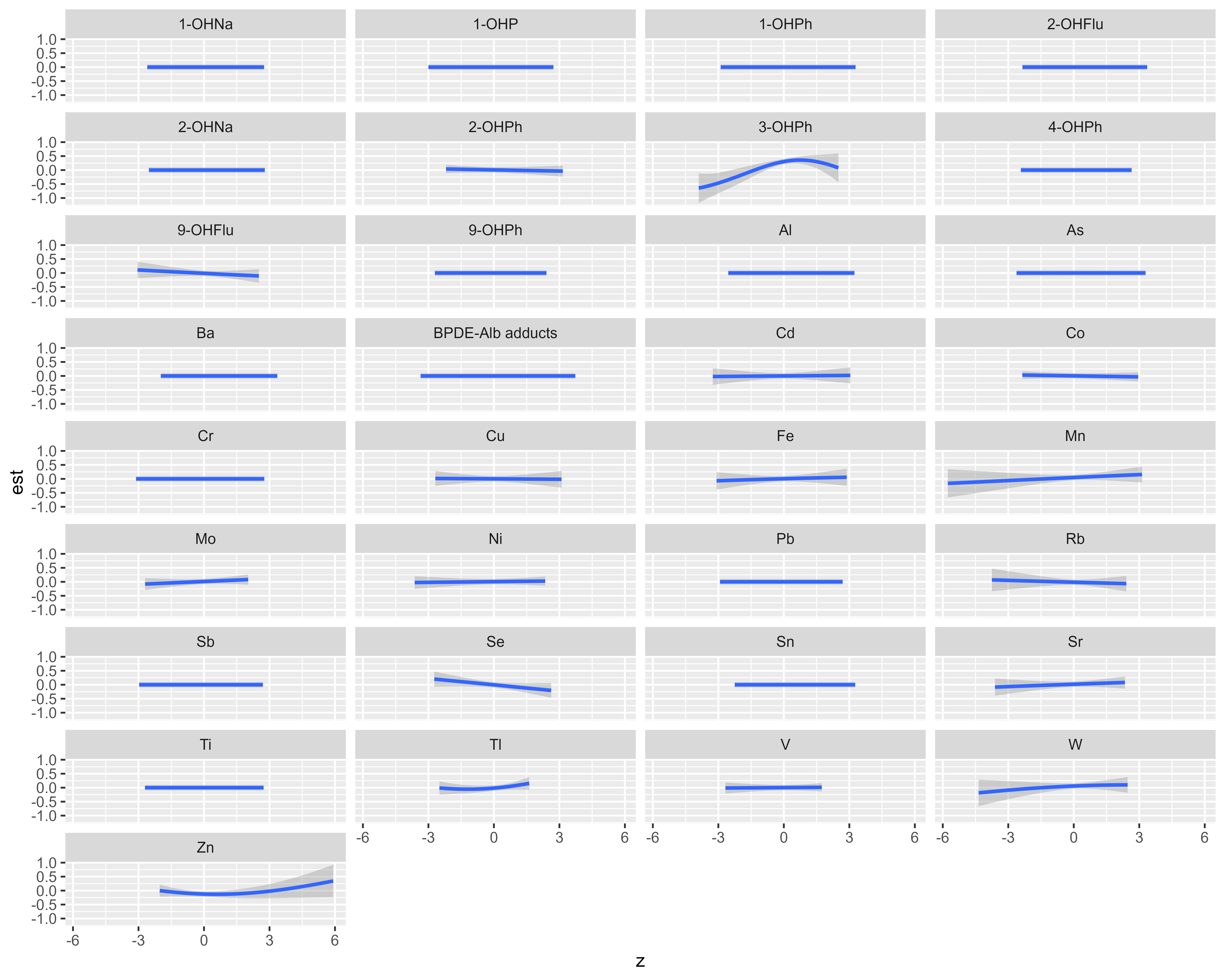


**Fig. S11.** Univariate exposure-response relationships between mixture and mtDNAcn in the BKMR model. The model was adjusted for age, sex, BMI, smoking status (current/non-current smoker), alcohol status (current/non-current drinker), TL at baseline, physical activity (yes/no), education level (junior high and below, senior high, college and above), and workplace (coke oven top, coke oven side/bottom, adjunct workplace, office).

**
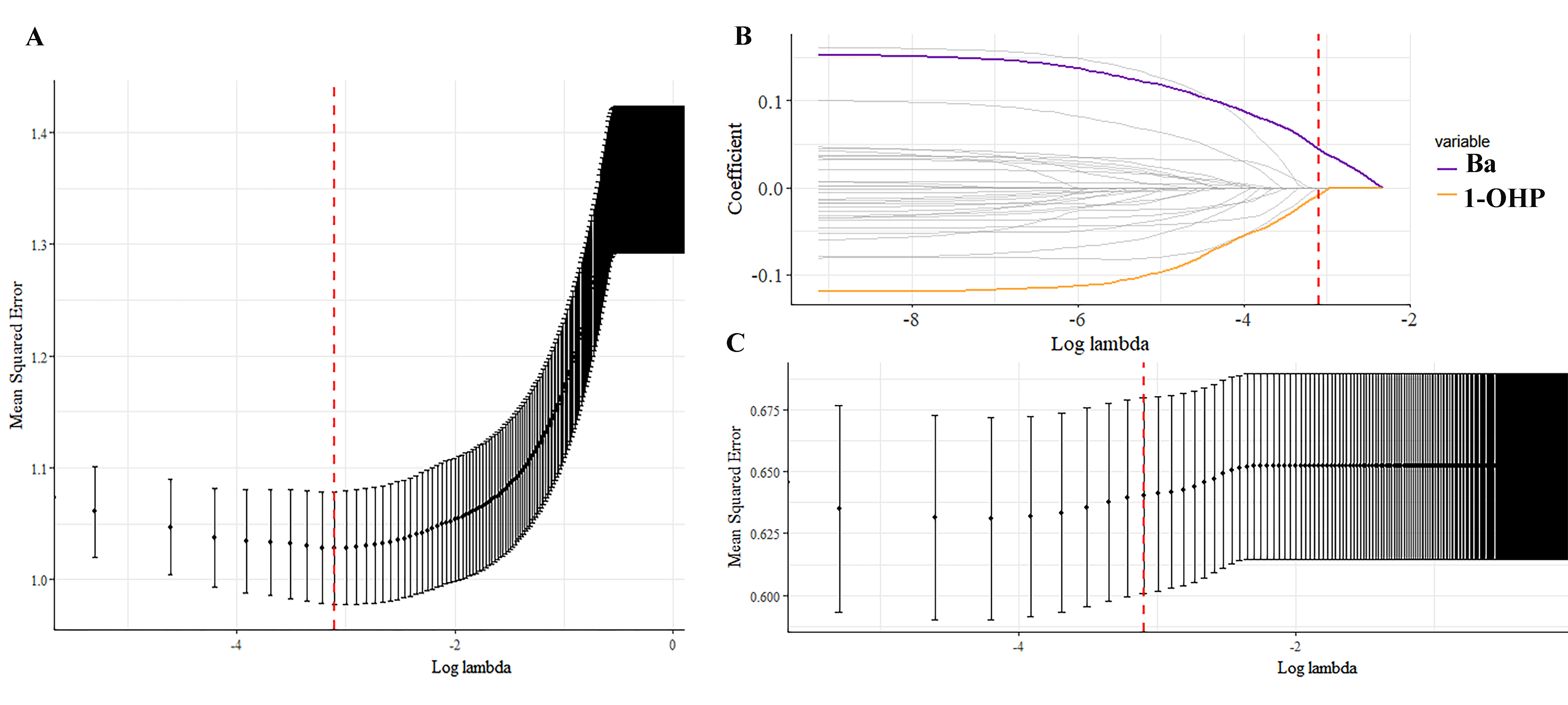
**

**Fig. S12.** The LASSO penalized regression analysis for the associations between mixture and aging biomarkers. (A) Cross-validation curve for the LASSO model with TL-ratio as the outcome. The red lines represented the value of ln (λ) with the minimum mean squared error (MSE). (B) Coefficient path plot for the LASSO model with mtDNAcn as the outcome. (C) Cross-validation curve for the LASSO model with mtDNAcn as the outcome. The vertical red dashed line indicates λ₁SE, the largest penalty within one standard error of the minimum-MSE λ, yielding the most parsimonious LASSO model with optimal predictive performance. Notes: The model was adjusted for age, sex, BMI, smoking status (current/non-current smoker), alcohol status (current/non-current drinker), TL at baseline, physical activity (yes/no), education level (junior high and below, senior high, college and above), and workplace (coke oven top, coke oven side/bottom, adjunct workplace, office). The black dotted line with its upper and lower standard deviation curves (error bars) represent the cross-validation curve along the λ sequence.


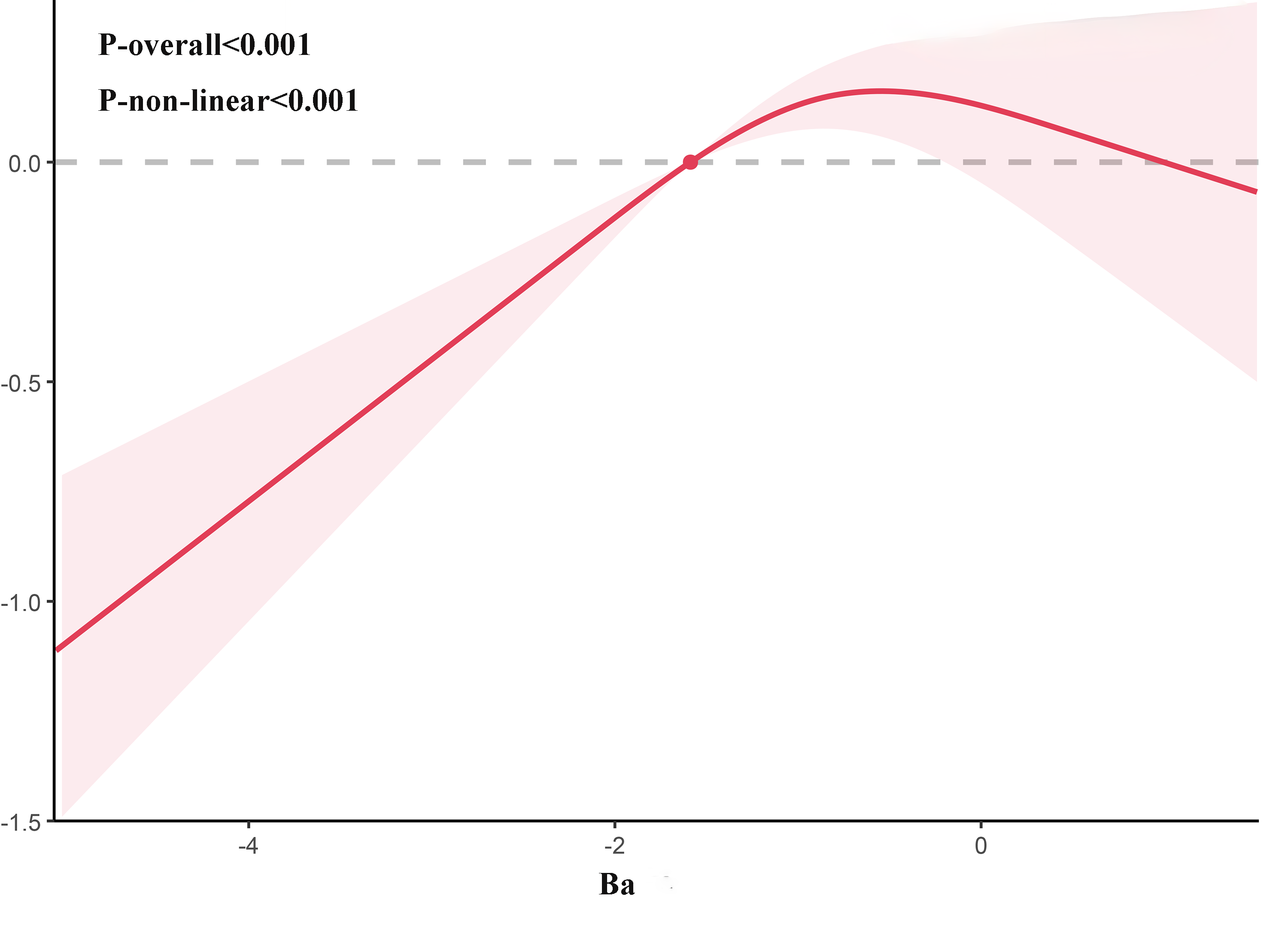


**Fig. S13.**The dose-response association of urinary PAHs and metals with mtDNAcn based on RCS regression model. Knots were placed at the 10th, 50th, and 90th percentiles of pollutants, and the reference value was set at the 50th percentile. Models were adjusted for age, sex, BMI, smoking status (current/non-current smoker), alcohol status (current/non-current drinker), TL at baseline, physical activity (yes/no), education level (junior high and below, senior high, college and above), and workplace (coke oven top, coke oven side/bottom, adjunct workplace, office). Solid lines indicate β, and shadow shape indicate 95 % CIs.
